# A comparative chemogenic analysis for predicting Drug-Target Pair via Machine Learning Approaches

**DOI:** 10.1038/s41598-020-63842-7

**Published:** 2020-04-22

**Authors:** Aman Chandra Kaushik, Aamir Mehmood, Xiaofeng Dai, Dong-Qing Wei

**Affiliations:** 1 Wuxi School of Medicine, Jiangnan University, Wuxi, China; 2 School of Life Sciences and Biotechnology, Shanghai Jiao Tong University, 800 Dongchuan Road, Shanghai, 200240 China

**Keywords:** Proteome informatics, Virtual drug screening, Computational models, Machine learning, Computational biology and bioinformatics, Genome assembly algorithms

## Abstract

A computational technique for predicting the DTIs has now turned out to be an indispensable job during the process of drug finding. It tapers the exploration room for interactions by propounding possible interaction contenders for authentication through experiments of wet-lab which are known for their expensiveness and time consumption. Chemogenomics, an emerging research area focused on the systematic examination of the biological impact of a broad series of minute molecular-weighting ligands on a broad raiment of macromolecular target spots. Additionally, with the advancement in time, the complexity of the algorithms is increasing which may result in the entry of big data technologies like Spark in this field soon. In the presented work, we intend to offer an inclusive idea and realistic evaluation of the computational Drug Target Interaction projection approaches, to perform as a guide and reference for researchers who are carrying out work in a similar direction. Precisely, we first explain the data utilized in computational Drug Target Interaction prediction attempts like this. We then sort and explain the best and most modern techniques for the prediction of DTIs. Then, a realistic assessment is executed to show the projection performance of several illustrative approaches in various situations. Ultimately, we underline possible opportunities for additional improvement of Drug Target Interaction projection enactment and also linked study objectives.

## Introduction

The accurate prediction of interactions formed between a drug and its targeted protein via computational approaches is highly demanding because it is an efficient analog to the wet-lab experiments that cost heavily and requires additional efforts. Drug–target interactions (DTIs) which are newly discovered are critical for discovering novel targets that can interact with the existing drugs, as well as new drugs that can target some specific genes causing diseases^1–3^. Drug repositioning is one of the efficient methods for the recovery of existing drugs for a novel cause, i.e. drugs which are developed for some particular purposes can be used to treat other biological conditions, meaning a single drug can be applied to many targets^4,5^. There is already massive research going on the existing drugs based on the bioavailability and their safe use. Repositioning can limit drug costs and may enhance the process of drug discovery, making drug repositioning an eminent method for drug discovery^6^. Some major techniques employed for the drug repurposing involve network-based approach^7^, network-based cluster approach^8^, network-based propagation approach^9^, text mining-based approach^10^, and semantics-based approach^11^. Drug repositioning is different from the traditional drug development that involves five stages, however, this method requires only 4 stages which include compound recognition, obtaining a compound, production and FDA based safety monitoring. The Gleevec (imatinib mesylate) is a well-known example of drug repositioning which was initially thought to interact only with the Bcr-Abl fusion gene related to leukemia. But later on, it was found that interaction of the Gleevec with PDGF and KIT can also be achieved, with an added advantage as a repositioned drug for the treatment of gastrointestinal stromal tumours^12,13^. The success of Gleevec as a repositioned drug is one of the admired stories reported in the literature^14–19^. As drug repositioning is already revealed by the example of Gleevec, it opens new doors for scientists to reposition other drugs as well. A drug’s feasibility (i.e. interaction of a single drug with multiple targets) may enrich its polypharmacology (i.e. having multiple beneficial effects), which motivates the scientists to discover more about drug repositioning.

On the other side, there still exist a lot of small molecules that can be used as drugs but because of their interaction profiles, they can not be used. For example, more than 90 million compounds are stored in the PubChem database whose interaction profiles are still unknown^20^. Thus, by knowing the interactions between the disease-causing genes and the target proteins for these compounds may help in the discovery of new drugs as it can help the drug candidates with low potential to work within the drug discovery field^21^. Likewise, the detection of various other interactions of this type may provide a deep understanding of the discovery of drug-targets that can have unwanted and adverse effects^22^. Therefore, for drug repositioning, the discovery of DTIs is very useful, as it aids with the drug candidate selection and predicts the side effects of these drugs in advance. Definitely, the experimental wet-lab techniques are more helpful in predicting such types of interactions but this job is much tiresome and also consumes a lot of time. Thus, from here, the computational methods take over as they are proven to be highly useful and may prove efficient in predicting potential interacting candidates with satisfactory accuracy, hence reducing the DTIs to be inspected via *in-vitro* correspondent.

AutoDock is a molecular docking platform that can model the flexibility in the targeted macromolecule optionally and protein-protein communications can be explored^23^. Based on the AMBER forcefield^24^, linear regression scrutiny and diverse protein-ligand complexes with identified inhibition constants, AutoDock has an improved free-energy scoring system.

The cmFSM is a parallel acceleration software available for classical frequent subgraph mining algorithm^25^. The main focus of this tool is to parallelize extension jobs by laboring parallel approaches. Simultaneously, it addresses the memory constraint issue as well by means of employing the multi-node approach. The mD3DOCKxb^26^ is designed on a coordinated parallel framework technique in which the collaboration of CPU and MICs attains elevated utilization of the hardware and is comprised of a new and efficient interaction engine that dynamically schedules the tasks.

SNPs have great importance in Genomics, Proteomics and precision medicine. One of the scalable and efficient tools is the mSNP that is an SNP identifying tool for a large-scale human genome that has availed a 38x single thread speedup on CPU, and zero loss in its accuracy, scaling up to 4,096 nodes^27^.

Another available platform is the A-CaMP^28^, which permits fast fingerprinting of the anticancer and antimicrobial peptides. It has robust coding architecture, has been developed in PERL language and is scalable with an accuracy of 93.4%.

The accuracy of sequence alignment also bears great importance. For multiple sequence alignments, the VCSRA^29^ (a Vector-based Center-star strategy-based algorithm using Suffix trees Recursively for multiple sequence Alignment) is a high duty platform that involves an elevated magnitude of parallelism. It is capable of carrying out the MSA in *O*(*mn* log2 *n*) time amid most alike sequences, where *m* is the number of sequences in a dataset and *n* refers to the sequences’ length average.

Virtual screening is used to search for possible potent hits that can be later confirmed through various docking and simulation analysis. One similar purpose efficient tool is the FlexX-Scan^30^ that is designed for an extremely fast, structure-based virtual screening, based on the incremental construction. It’s a compact descriptor for showing favorable protein interaction points.

In the present time, mainly there are three main approaches related to the computational methods for discovering DTIs. The first one is the ligand-based approach, which is based on the concept that molecules with similar properties usually share their properties and binds with the same kind of proteins^31^. In general, the interactions are predicted by using the fact of similarity between the proteins and ligands^32^. In case of the less number of reported ligands per protein, the result of the ligand-based approach may be ambiguous^33^.

The second approach is the docking approach, a 3D structure of the drug and a protein is taken and then a simulation program is run to determine whether they can interact or not^34–37^. However, some proteins with unknown 3D structures are there to which docking cannot be applied. Some of the membrane proteins in drug targets^38^ are challenging to predict their 3D structure^39^. Furthermore, protein flexibility can also be one of the challenging factors while dealing with a receptor protein, as we require a certain degree of freedom, so that exact calculations can be carried out.

The third approach is the chemogenomic approach. Here, the prediction is carried out by collecting the information from both drugs and targets. The chemogenomic approach is associated with the advantage of working with extensively abundant biological data for prediction. The chemical structures’ charts and nucleotide sequences for the drugs and targets are widely used as information while predicting DTIs^40^ and can be easily obtained from the publicly available online databases. Some of the challenges that need to be addressed regarding this new technique are the requirement of an additionally enhanced refined integration of bioinformatics and chemoinformatics information, selection of top compounds from the existing infinite artificial possibilities by a more rational technique and to be able to construct additional catalogs that are information specific^41^.

In this investigation, the more popular chemogenomic methods are being revised. The investigation initiated by knowing different types of data required to perform the prediction task and finding the source of data along with exploring ways to use the same data in prediction.

After comparing with the reported literature on the DTI prediction approach^1,2,5,42,43^, our survey is found to be more comprehensive and closely related to the already existing chemogenomic methods for the prediction of DTIs. Moreover, a novel approach is provided in this work for the categorization of various chemogenomic methods. Furthermore, various kinds of data have been described here that is being used for the chemogenomic prediction tasks; however, our focus was mainly on the software listing packages that produce various characteristics in demonstrating drugs and targets (conflicting with online databases available for the information on DTIs)^44^.

The latest review presented by Chen *et al*.^2^ describes a complete online database that stores all the information related to drugs and their targets ((KEGG)^45^ and (DrugBank))^46^. Along with the algorithms, online web servers were described for the prediction of interactions and the discussions over the drug identification are carried thoroughly. The aim of our investigation is comparable to the work reported by Chen *et al*. in terms of reviewing the state-of-the-art methods and to deliver potential future direction in this field of research. However, we have categorized different prediction methods very precisely and also suggest different directions towards future research, significantly different from those reported by Chen *et al*.

## Materials and Methods

### Interaction data

This type of data can be found on several publicly accessible online databases that keep a record of particular targets and their drugs. Some of the repositories employed for this work include KEGG^45^, DrugBank^46^, ChEMBL^47^, and STITCH^48^. The data collected on interaction from these databases is usually configured in the form of a linkage medium among the targets and their drugs. This medium match up with the bipartite graph where drugs and targets are represented by nodes, and in the form of edges, connecting drug-target pairs interaction^3,49^.

### Nearest profile and weighted profile

Two methods introduced by Yamanishi *et al*.^40^ are the Nearest Profile and Weighted profile. The nearest profile is the linking outline for a novel drug or target with its nearest neighbor (i.e. the most similar drug or target to the drug). For instance, to calculate a nearby outline for a new drug *d*_*i*_, we follow:

1 $$\hat{Y}({{d}}_{i})={S}_{d}({{d}}_{i},{{d}}_{nearest})\times Y({{d}}_{nearest}).$$

Here $Y({{d}}_{i})$ denotes the interaction profile of the drug ${{d}}_{i}$ and ${{d}}_{nearest}$ denotes the drug that resembles the ${{d}}_{i}$ the most. However, in the Weighted Profile section; we use all the similarities of different drugs or targets and calculate a weighted average for them. The calculation of the weighted profile for drug ${{d}}_{i}$ is done using:

2 $$\hat{Y}({{d}}_{i})=\frac{{\sum }_{j=1}^{n}\,{S}_{d}({{d}}_{i},{{d}}_{j})\times Y({{d}}_{j})}{{\sum }_{j=1}^{n}\,{S}_{d}({{d}}_{i},{d}_{\dot{j}})}$$

We calculated the average of the forecasts from the drug and the target to gain the ultimate estimates.

### Regularized least-squares with weighted nearest neighbors

The other technique which was founded on RLS-Kron^50^ was introduced in^51^, where the performance of RLS-Kron was increased with a preprocessing technique WNN having the same as that of NII. WNN can be used to deduce an interaction profile for every new drug *d*_*i*_,:

3 $$Y({{d}}_{i})=\mathop{\sum }\limits_{J=1}^{n}\,{\omega }_{j}Y({{d}}_{j}),$$

Based on similarity to drug d_j,_ the drugs d_i_ to d_n_ are arranged in descending order and ${\omega }_{j}={\eta }^{j-1}$ where $\eta$ denotes the decay term and $\eta \le 1$. This procedure is applied from the target side also, and then the RLS-Kron method is used as a usual process. By applying the WNN method with NII, the prediction performance boost up which shows that these preprocessing methods performed well.

### Network-based inference

Network-based inference (NBI)^52^ applies network diffusion on the DTI bipartite network corresponding to the linkage matrix *Y* to perform predictions. The working of network diffusion follows:

4 $$\hat{Y}=WY,$$

Where $W\in {{\mathbb{R}}}^{n\times n}$ is the weight matrix can be defined as:

5 $${W}_{ij}=\frac{1}{{{\Gamma }}_{(i,j)}}\mathop{\sum }\limits_{l=1}^{m}\,\frac{{Y}_{il}{Y}_{jl}}{k({t}_{l})}$$

Where *Γ* is the diffusion rule. Whereas, k(x) denotes the degree of node i.e., x in the DTI bipartite network. In the NBI case, the *Γ* rule is given by:

$${\Gamma }=k({{d}}_{j}).$$

### Kernelized bayesian matrix factorization with twin kernels

Kernelized Bayesian Matrix Factorization with Twin Kernels (KBMF2K)^53^ in our view, is the first method to use matrix factorization for the prediction of DTIs. It employes a Bayesian probabilistic design along with the concept of matrix factorization to complete the forecast. In other words, nonlinear dimensionality reduction is performed by the use of variational approximation and, hence the efficiency of computation time taken by this method has been improved. The algorithmic details of this method are very broad, so a negligible impression of the algorithm is provided here^53^.

### Collaborative matrix factorization

Collaborative Matrix Factorization (CMF)^54^ practices cooperative filtering for forecasting. The key purpose of matrix factorization is to discover two matrices *A and B* where $3A{B}^{T}\approx Y$, while CMF proposes regularization terms to guarantee that $A{A}^{T}\approx {S}_{d}$ and $B{B}^{T}\approx {S}_{t}$. The objective function for CMF is given by $\mathop{{\rm{Min}}}\limits_{A,B}{\Vert W\otimes (Y-A{B}^{T})\Vert }_{F}^{2}+{\lambda }_{l}({\Vert A\Vert }_{F}^{2}+{\Vert B\Vert }_{F}^{2})+$

6 $${\lambda }_{d}{\Vert {S}_{d}-A{A}^{T}\Vert }_{F}^{2}+{\lambda }_{t}{\Vert {S}_{t}-B{B}^{T}\Vert }_{F}^{2}$$

where ${\Vert .\Vert }_{F}$ is the Frobenius norm, ⊗ is the elementwise product, ${\lambda }_{l},{\lambda }_{d},\,and\,{\lambda }_{t}$ are parameters and $W\in {{\mathbb{R}}}^{n\times m}$is weight matrix where *W*_*ij*_ = 0 for unknown drug-target pairs, so that in the estimation of *A and B* they have no role. The first line is the weighted low-rank approximation that tries to reconstruct *Y* by finding the latent feature matrices *A and B*. The second line is the Tikhonov regularization term that provides simpler solutions by preventing the larger values and helps in avoiding overfitting. The 3^rd^ and 4^th^ ranks are normalization terms that require latent feature vectors of similar drugs/targets to be similar and latent feature vectors of unlike drugs/targets to be dissimilar correspondingly.

MSCMF is another variant of CMF which involve the use of multiple similarities for both the drug and the target^54^. Rather than the chemical structure similarity and genomic sequence similarity that is typically used for the drugs and targets respectively. ATC similarity is also used for drugs, and GO and PPI network similarities are used for the targets. The MSCMF objective function is given as:

7 $$\begin{array}{c}\mathop{{\rm{\min }}}\limits_{A,B}{\Vert W\otimes (Y-A{B}^{T})\Vert }_{F}^{2}+{\lambda }_{l}({\Vert A\Vert }_{F}^{2}+{\Vert B\Vert }_{F}^{2})+{\lambda }_{d}{\Vert \mathop{\sum }\limits_{k=1}^{{M}_{d}}{\omega }_{d}^{k}{S}_{d}^{k}-A{A}^{T}\Vert }_{F}^{2}\\ +{\lambda }_{t}{\Vert \mathop{\sum }\limits_{k=1}^{{M}_{t}}\,{\omega }_{t}^{k}{S}_{t}^{k}-B{B}^{T}\Vert }_{F}^{2}+\,{\lambda }_{\omega }({\Vert {\omega }_{d}\Vert }_{F}^{2}+{\Vert {\omega }_{T}\Vert }_{F}^{2})\end{array}$$

s.t. $|{\omega }_{d}|=|{\omega }_{t}|=1$ where ${M}_{d}\,and\,{M}_{t}$ represent the number of drugs and targets’ similarity matrices respectively and ${\lambda }_{\omega }$ is a parameter. The ${\omega }_{d}\,and\,{\omega }_{T}$ are the weight vectors for the linear combination of similarity matrices of drugs and targets respectively. Tikhonov regularization terms for ${\omega }_{d}\,and\,{\omega }_{T}$, while the sixth term is a restriction that ensures that weight of ${\omega }_{d}\,and\,{\omega }_{T}$ sum up to 1.

### Weighted graph regularized matrix factorization

Weighted Graph Regularized Matrix Factorization (WGRMF)^55^ is similar to CMF except that it practices chart normalization terms to learn a manifold for label propagation. The objective function for WGRMF is given as:

8 $$m{\rm{i}}{n}_{A,B}{\Vert W\otimes (Y-A{B}^{T})\Vert }_{F}^{2}+\,{\lambda }_{l}({\Vert A\Vert }_{F}^{2}+{\Vert B\Vert }_{F}^{2})+{\lambda }_{d}{T}_{r}({A}^{T}{\tilde{l}}_{d}A)+{\lambda }_{t}{T}_{r}({B}^{T}{\tilde{l}}_{t}B)$$

where $\,{T}_{r}(.)$ is the trace of the matrix, and ${\tilde{l}}_{d}\,and\,{\tilde{l}}_{t}$ are the normalized graph Laplacians which are obtained from ${S}_{d}\,and\,{S}_{t}$ respectively. ${S}_{d}\,and\,{S}_{t}$are sparsified before calculating the Laplacians graph via having only a pre-selected value of closed neighbors for individual drug and its target respectively. For more details on the graphical regularization please refer to^56,57^.

The role of the weight matrix is the same as in the CMF; we can control that unknown drug-target pair don’t contribute to interactions’ prediction by setting ${W}_{ij}=0$. The weight medium is vital as or else the test cases would sum no interactions (i.e. negative instances) and have unwanted effects on the predictions; for more information, refer to the available supplementary data.

## Results

### Drug and target data classifiers

The data available for a different type of drugs can be used to train new DTI classifiers but the available information must not be limited only to the graphical representations, including chemical structures^58^, side effects^59^, Anatomical Therapeutic Chemical (ATC) codes^60^, and how genes respond to different types of drugs^61^. Data can be obtained in many useful forms from the chemical assembly charts of drugs which also includes substructure fingerprints in addition to the constitutional, topological and geometric signifiers among other molecular characteristics (e.g. via the Rcpi^62^, PyDPI^63^ or Open Babel^64^ packages). The available data that can be obtained for the targets include genomic sequences^65^, Gene Ontology (GO) information^66^, gene expression profiles^67^, disease associations^68^ and protein-protein interaction’s (PPIs) network information^69,70^ among others. Moreover, additional data for the targets are obtained as well from the amino acid sequences, that involves its arrangement, CTD (composition, transition, and distribution) and auto correlativity signifiers (e.g. via the PROFEAT Web server^71^).

In the past few years, many (chemogenomic) DTI prediction methods have been developed^50,51,54–57,72–104^. Based on different techniques, these methods are employed for the prediction, which briefly explains and categorizes them according to the techniques employed.

Neighborhood Weighted Profile, Bipartite local models, Network diffusion and Matrix factorization, the supplied information is used in these techniques, comprising of a linking matrix $Y\in {{\mathbb{R}}}^{n\times m}$ that displays the interacting drugs and targets, a drug similarity matrix ${S}_{d}\in \,{{\mathbb{R}}}^{n\times n}$ and a target similarity matrix ${S}_{t}\in \,{{\mathbb{R}}}^{m\times m}$. While in the ‘feature-based classification’ section, the similarity matrices both for the drug and target have been replaced by feature matrices, ${F}_{d}\in {{\mathbb{R}}}^{n\times p}\,and\,{F}_{t}\in {{\mathbb{R}}}^{m\times q}$ which represents the drugs and targets respectively.

### Empirical evaluation

Here we have done a broader empirical evaluation among various methods, under three different CV settings:

1. S1, where some arbitrary pairs are left out of the test set.
2. S2, where complete drug profiles are left out of the test set
3. S3, where complete target profiles are left out of the test set.

S1 is a traditional setting for assessment. However, S2 and S3 are proposed to assess the capability of various methods to predict novel drug and target interactions. Here, novel drugs and targets are those for which no interaction information is available. Besides, the experiments conducted under the S2 and S3 draws a complete picture of how the performance of different methods differ according to various situations.

The results of the different methods under the CV settings have already been visualized in Figs. 2 and 3. All the outcomes of this study are explained, including their advantages and disadvantages for each of the methods along with other general observations. It is worthy to note that results on the NR data set were found inconsistent probably due to its smaller size^43^.

**Figure 1:**
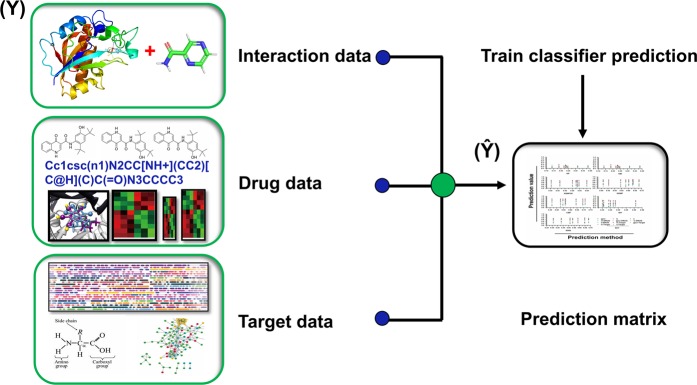
Flowchart of DTI prediction task using a chemogenomic prediction. Three different types of data have been used for the DTIs prediction.

**Figure 2:**
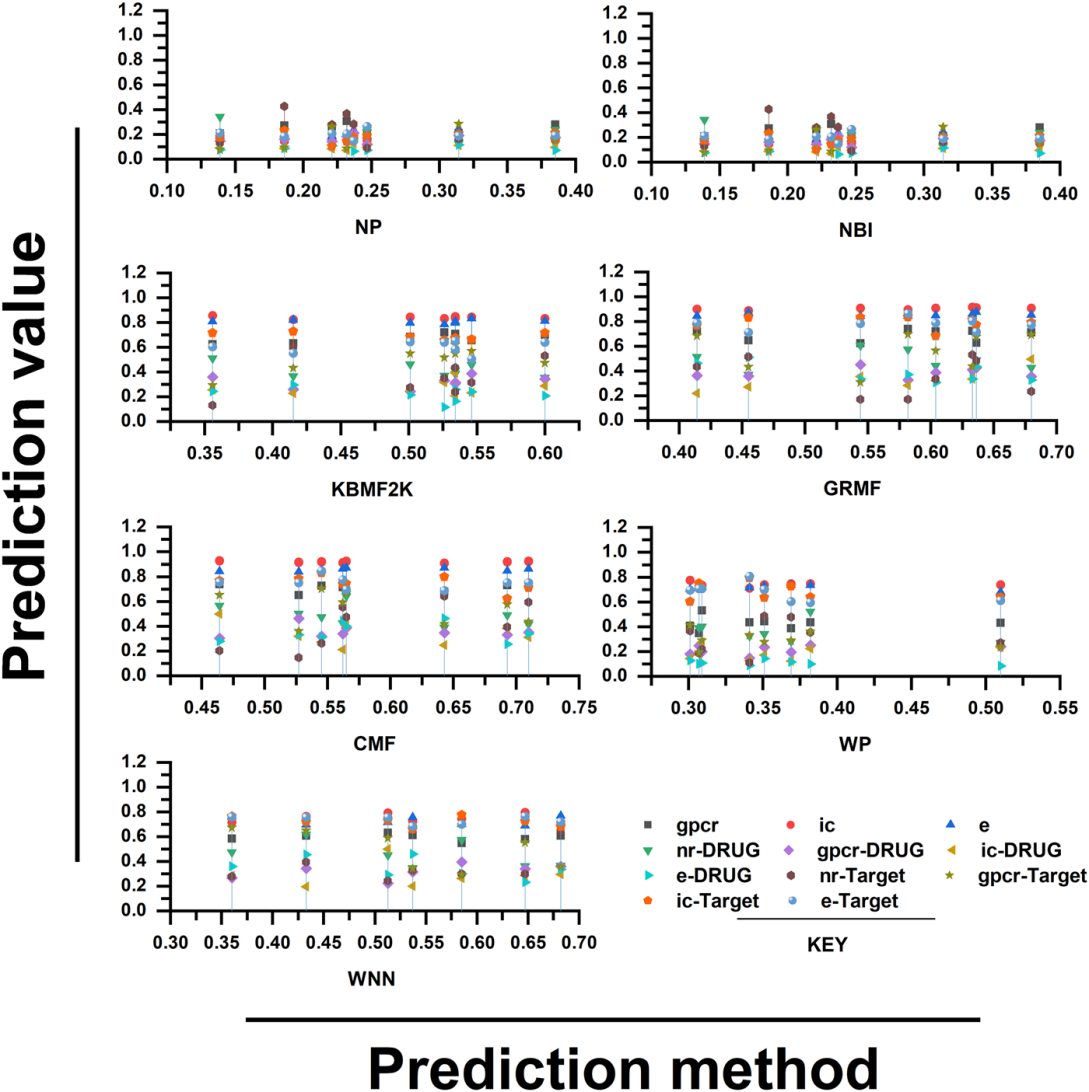
Depicts the DTIs prediction using different methods, X-axis represents the applied methods and Y-axis indicates the DTIs’ prediction scores. All the methods (NP (Nearest Profile), NBI (Network-based inference), KBMF2K (Kernelized Bayesian Matrix Factorization with Twin Kernels), WGRMF (Weighted Graph Regularized Matrix Factorization), CMF (Collaborative Matrix Factorization (CMF), WP (Weighted Profile), and WNN (Weighted Nearest Neighbors)) show approximately same prediction score with minor changes except WGRMF that achieved comparatively highest value. The NP and NBI approach exhibits comparatively much lower prediction scores.

**Figure 3:**
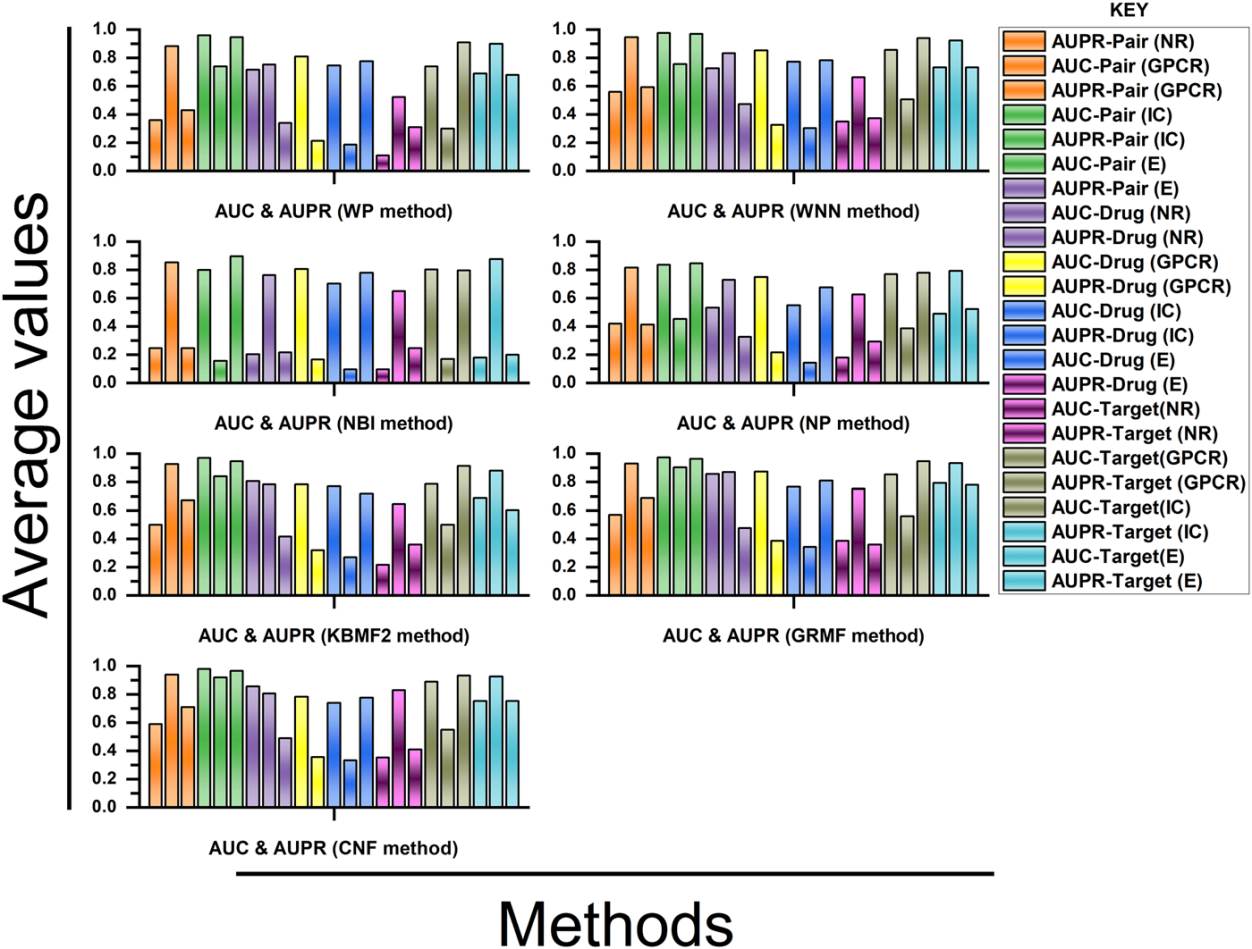
Depicts the AUC (Area Under the Curve) and AUPR (Area Under the Precision-Recall) scores using different methods, X-axis represents the applied methods and Y-axis indicates the average AUC and AUPR scores of DTI.

### Pair prediction case (Drug-target interaction)

**Figure 4:**
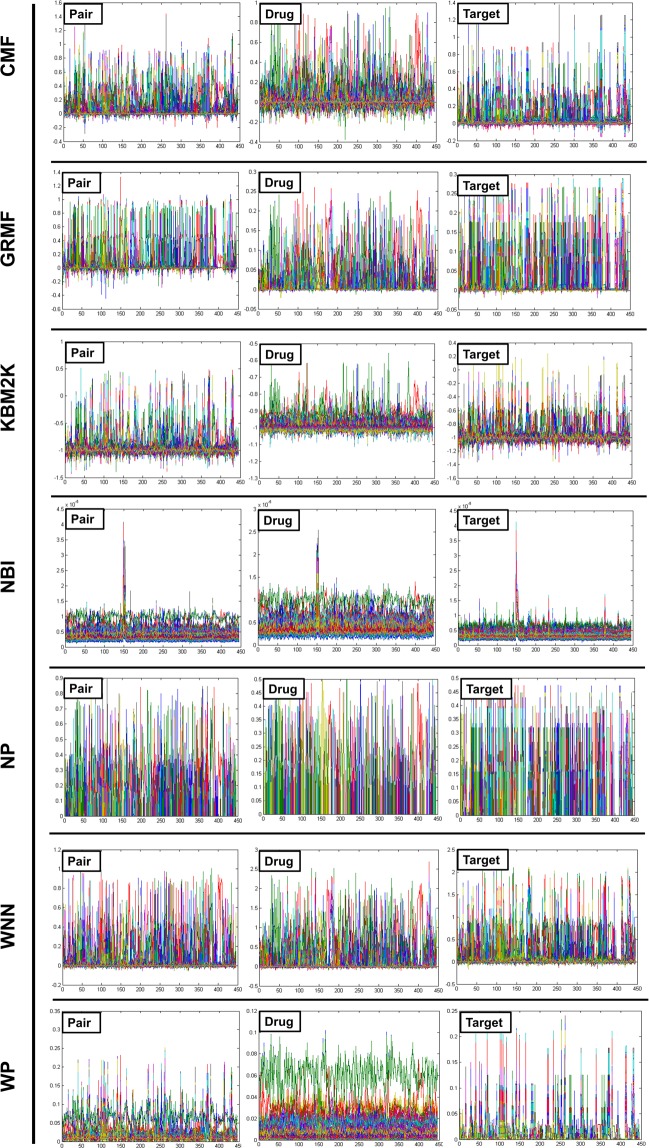
The different cross-validation settings: 1: Pair (DTI)- involves drug-target pairs from the interaction matrix Y to use as the test set, 2: Drug- is the setting where entire drug profiles are shown and 3: Target- entire target profiles. The CV settings for S1, S2, and S3 are provided on the X-axis while the Y-axis represents the standard deviation (SD) of all the employed techniques.
(ii) In the ion channels (IC) and enzymes (E) data sets, the performance of the Weighted Profile is better than the Nearest Profile. This is due to the reason that IC and E data sets are larger than non-redundant (NR) and G-Protein Coupled Receptor (GPCR) counterparts having a large number of neighbors. Therefore, interactions can be deduced more accurately (Fig. 4).

Based on the results obtained from Figs. 2 and 3, the following two conclusions have been made:

(i) Under the DTI CV settings, CMF is found to be the best method, followed by WGRMF. It means that the matrix factorization method is finest over other methods, which makes them the most promising DTIs prediction methods for the study of DTIs (Fig. 4).

### Drug prediction case (Drug)

Ongoing from the drug-target interaction CV setting to the Drug CV setting, it was observed that the results in Fig. 4 were more interesting than the Drug-target interaction. Usually, it is more difficult to predict interactions for the drugs or targets which are unknown in the test sets. This is different from the Drug-target interaction where the drug or target interaction profiles are partially missing out.

The performance of WGRMF is best, followed by the CMF. Therefore, the Matrix Factorization method is again performing well in general. The WGRMF has done well than the CMF under Drug setting because of its graph regularization terms. This also expresses the benefits of manifold learnings while it is an informative locale.

RLS-WNN, which is based upon the network similarities also provides a useful prediction performance. The reasonable performance of RLS-WNN is due to its preprocessing procedure which strengthens its learning progression by inferring to the temporary profiles for the missing drugs. The network similarity in RLS-WNN is calculated by the GIP kernels which can be used in the algorithm later on. Logically, temporary profiles are indeed better for calculating network similarity than the initially empty profiles of the missing drugs, which underlines the significance of preprocessing procedures like WNN when the inclusion of a network similarity in training the classifiers is intended.

### Target prediction case (Target)

As projected, the AUPR (Area Under the Precision-Recall) results of the Target settings are relatively lower than the S1 setting but are gradually higher than those of the results obtained under drug-target interaction settings. Methods including Matrix Factorization are usually better in drug cases. From here, we conclude that the target genomic sequence similarities are extremely better even than the similarities of drugs’ chemical structures. The performance of WGRMF is better even than the CMF due to the involvement of graph regularization terms. However, RLS-WNN has an average performance. As for NBI, similar to the Drugs’ cases and Drug-target interactions, It is not capable to outperform the Nearest Profile, baseline methods, and Weighted Profiles. Therefore, it is concluded that the best choice for the prediction of DTIs is network-based methods as shown in Fig. 4; for more information (Supplementary Data).

## Discussion

Many computational techniques are involved in drug repositioning which is used in various conditions, depending on the existing knowledge about the concerned disease or adverse condition. Using these methods, we have generated an outline of DTI prediction, which is an important aspect of the drug discovery process. Many web servers have been developed to deal with this work for practitioners, intending to perform this work on a universal scale.

Generally, in the prediction of DTIs, the best method reported is the Matrix Factorization method. In addition to this, the manifold assumption is that the point lies on or near to the low dimensional manifold^90–92^ are more successful for the improvement of DTIs’ prediction performance (as demonstrated by WGRMF). It is essential to state that the RLS-WNN method did not compete with the Matrix Factorization method in the DTIs prediction but an added advantage is the faster algorithm (RLS-WNN). However, when someone wants to predict DTIs, it is beneficial to obtain the primary predictions by RLS-WNN first. It is also highlighted that if the data sets are larger, then the BLMs (Bipartite local models) are the best to be considered as they are proved to be faster and efficient.

While considering the network-based method (NBI), it did not perform well in comparison to other methods which may be due to the properties of DTIs networks that are not satisfactory to deal with network-based methods. Examples related to the interactions of drugs or targets present in the network are very less or there may be the presence of undiscovered interactions present in the noninteracting groups (which may have a negative influence upon the obtained prediction). Moreover, their performance in the prediction of new interactions for orphan drugs (previously unknown interactions) is not well discovered. However, this problem becomes more complex when attempts are being made to predict new interactions for the orphan targets as well; this is because of the indirect network path between the orphan drug and its target which gives a low prediction score; for more information (Supplementary Data).

## Conclusion

Alternatively, network-based methods still have a significant role in predicting DTIs. For example, the NRWRH^80^, the generation of a heterogeneous network is a prominent idea for performing DTIs prediction. By improving the heterogeneous network with more data (i.e. addition of more drug-target pairwise similarities) can help the network-based methods to solve the issues occurring in DTIs prediction for orphan drugs or targets up to some extents. It is also helpful to be inspired from the previous effort on generating functional linkage network (FLNs). FLNs are functionally linked networks between genes that have been used successfully in genes-related functions and disease research. To construct FLN, it requires the information collected from various heterogeneous resources of varying classes and comprehensiveness that may highly correlate with each other. Such understanding in creating FLNs can be delivered to the generation of heterogeneous DTI networks on which network-based methods can be applied for new DTIs prediction with greater precision and accuracy.

In the present work, we have started with a brief description of the data that we required for the drug-DTI prediction and also showed some examples that could be used for its prediction. An outline of different methods is given that are trained with the available data. After this, we have performed an empirical comparison between the methods which are best in their respective category, to illustrate their prediction performances under different situations. At last, a compiled list of all the possibilities was provided for further enhancement of the prediction performance.

According to data, the datasets are binary in nature, i.e. given an interaction matrix *Y* (where *Y*_*ij*_ = 1 if the drug and target interact with each other, if there is no interaction *Y*_*ij*_ = 0); that creates another possibility. Some of the interactions where *Y*_*ij*_ = 0 have not yet been discovered, which may create a problem in the training process for various classifiers. Besides, there is another possibility that in a real situation, the drug-target pairs having binding energies, showing variations over a wide range of the spectrum (interactions are not binary on/off). Some data sets having continuous values representing drug-target binding energies (as opposed to distinct 0 and 1 values). For that reason, using such continuous-valued data sets is more useful because it represents the actual situation than the binary sets in a better way which has been used earlier in the DTI’s prediction extensively.

### Future direction

The type of work mentioned above particularly focuses on the target proteins, but there is another type of target which is the noncoding RNAs (ncRNAs), and the drugs which are successfully developed. These are the RNAs that are not protein-coding, and they contain subcategories which include microRNAs (miRNAs), long coding RNAs (lcRNA) and Intronic RNAs (iRNA) among several others. A few examples are the use of miRNAs to treat the Hepatitis C virus and Alport nephropathy. The behavior and mechanism of each of the ncRNAs are quite. Research on chemogenomic methods for prediction of ncRNAs is likely to continue for the next several years with contributions involving deep learning concepts, Multiview learning and possibly unprecedented clever features for representing drugs or targets. Therefore, it leads to different opportunities and challenges, all of which are discussed with examples in the recent reports regarding DTIs.

## Supplementary information

Supplementary information.

## Data Availability

The way we want to predict the new DTI is completely different from the existing training data. The data which represents the drug and the target involved in the interaction is also needed for this purpose. The overall workflow for the prediction of new DTIs is graphically produced (Fig. 1). Interaction data were retrieved from different sources. Drugs data were retrieved from Rcpi, PyDPI, and Open Babel. Targets data were retrieved from Gene Ontology (GO) information, gene expression profiles, gene sequence, disease associations, and protein-protein interaction (PPI) network information; for more information (Supplementary Data). All data generated and analyzed during this study are included in this article. The proposed DTI (Dataset, Statistical Metrics, Confidence Interval & Benchmark Evaluation Results) is freely accessible at http://weislab.com/WeiDOCK/?page=DTI. The provided data includes dataset files (.txt format), metrics files (.mat format), statistical metrics (.mat format), confidence intervals (.mat format), benchmark evaluation results (.mat format), and scripts for executing this DIT Model (.py format).
