# Supplementary material for: A comparative chemogenic analysis for predicting Drug-Target Pair via Machine Learning Approaches
Source: Sci Rep. 2020 Apr 22;10:6870. doi: 10.1038/s41598-020-63842-7 (PMC7176722; doi:10.1038/s41598-020-63842-7)
Supplement: Supplementary file 1 — Supplementary information. [file 41598_2020_63842_MOESM1_ESM.docx]

# A comparative chemogenic analysis for predicting Drug-Target Pair via Machine Learning Approaches

^*^Aman Chandra Kaushik^1,2^, Aamir Mehmood^2^, Xiaofeng Dai^1^, and ^*^Dong-Qing Wei^2^

^1^Wuxi School of Medicine, Jiangnan University, Wuxi, China, ^2^School of Life Sciences and Biotechnology, Shanghai Jiao Tong University, 800 Dongchuan Road, Shanghai 200240, China

**Authors’ information**

Emails: [amanbioinfo@jiangnan.edu.cn](mailto:amanbioinfo@jiangnan.edu.cn); [aamirmehmood@sjtu.edu.cn](mailto:aamirmehmood@sjtu.edu.cn); [xiaofeng.dai@jiangnan.edu.cn](mailto:xiaofeng.dai@jiangnan.edu.cn); dqwei@weislab.com

***Correspondence:**

^*^**Aman Chandra Kaushik**

Ph.D., Professor, School of Medicine, Jiangnan University. [amanbioinfo@jiangnan.edu.cn](mailto:amanbioinfo@jiangnan.edu.cn)

1800, lihu avenue, wuxi, jiangsu, 214122; Tel: 86-15-61898-7739

^*^**Dong-Qing Wei**

Dong-Qing Wei, Ph.D., Professor, Department of Bioinformatics and Biostatistics College of Life Sciences and Biotechnology, The State Key Laboratory of Microbial Metabolism,

Shanghai Jiao Tong University. [dqwei@sjtu.edu.cn](mailto:dqwei@sjtu.edu.cn), 800 Dongchuan Road Shanghai, Minhang District China, 200240 Tel: 86-21-3420-4573

**SUPPORTING INFORMATION**

# Materials & Methods

# Data availability and datatypes

The way we want to predict the new drug and target interaction is completely different from the existing training data. The data which represents the drug and the target involved in the interaction is also needed for this purpose. The data required is described below in a more detailed view (Figure 1).

## Interaction data

This type of data can be found on several publicly accessible online databases that keep a record of particular targets and their drugs. Some of the repositories employed for this work includes KEGG [^1^](#_ENREF_1), DrugBank [^2^](#_ENREF_2),ChEMBL[^3^](#_ENREF_3)and STITCH [^4^](#_ENREF_4). The data collected on interaction from these databases is usually configured in the form of a linkage (connection) medium among the targets and their drugs. This medium match upwith the bipartite graph where drugs and targets are represented by nodes, and in the form of edges which connects drug-target pair interactions.

## Drug and target data

##

## Data available for different type of drugs can be used to train new DTI classifiers but the data must not be limited to graphical representation which includes chemical structure [^5^](#_ENREF_5), side effects [^6^](#_ENREF_6), Anatomical Therapeutic Chemical (ATC) codes [^7^](#_ENREF_7)and how genes respond to different types of drugs[^8^](#_ENREF_8). Data can be obtained in many useful forms from the chemical assembly charts of drugs which also includes substructure fingerprints in addition toconstitutional, topological and geometric signifier among other molecular characteristics (e.g. via the Rcpi [^9^](#_ENREF_9), PyDPI [^10^](#_ENREF_10)or Open Babel[^11^](#_ENREF_11)packages). The available data that can be obtained for the targets includes genomic sequences [^12^](#_ENREF_12), Gene Ontology (GO) information [^13^](#_ENREF_13), gene expression profiles [^14^](#_ENREF_14), disease associations [^15^](#_ENREF_15)and protein-protein interaction (PPI) network information [^16^](#_ENREF_16)^,^[^17^](#_ENREF_17)among others. Moreover, additional data for the targets are obtained as well from amino acid sequences, that’s involves its arrangement, CTD (composition, transition and distribution) and autocorrelativity signifiers (e.g. via the PROFEAT Web server [^18^](#_ENREF_18)).

In the past few years, many (chemogenomic) DTI prediction methods have been developed [^19-57^](#_ENREF_19). On the basis of different techniques these methods are employed for prediction, we have briefly explained to and categorized them according to the techniques employed.

In further subsections namely ‘Neighborhood’, ‘Bipartite local models’, ‘Network diffusion’ and ‘Matrix factorization’, the supplied information is used in these technique, comprising of a linking matrix $Y\in\mathbb{R}^{n\times m}$ which displays the drugs and targets that interact, a drug similarity matrix $S_{d}\in\mathbb{R}^{n\times n}$and a target similarity matrix $S_{t}\in\mathbb{R}^{m\times m}$. While in‘feature-based classification’ section the similarity matrices both for drug and target has been replaced by feature matrices, $F_{d}\in\mathbb{R}^{n\times p}and F_{t}\in\mathbb{R}^{m\times q}$which represents the drugs and targets respectively.

**Neighbourhood Weighted Profile**

Neighbourhood methods perform predictions on the basis of functions that are relatively simple. More accurately, on the basis of previous similarities and interaction data for drugs and targets, its linking outline can be obtained for any novel target and hier respective drugs. By a novel drug, we mean the drug whose target is unknown and likewise a new target is the one with unknown drugs or pre-identified interactions.

**Nearest profile and weighted profile**

Two methods that were introduced in this paper [^58^](#_ENREF_58) are Nearest Profile and Weighted profile methods. The nearest profile is the linking outline for a novel drug or target with its nearest neighbour (i.e. the most similar drug or target to the drug). For instance, to calculate a nearby outline for a new drug *d_i_*,we follow:

$\hat{Y}\left( ⅆ_{i} \right)=S_{d}\left( ⅆ_{i},ⅆ_{nearest} \right)\times Y(ⅆ_{nearest})$. (1)

Here $Y\left( ⅆ_{i} \right)$ denotes the interaction profile of the drug $ⅆ_{i}$ and $ⅆ_{nearest}$ denotes the drug that resembles the $ⅆ_{i}$ most. However, in Weighted Profile section; we use all the similarities of different drugs or targets and calculate a weighted average for them. The calculation of weighted profile for drug $ⅆ_{i}$ is done using:

$\hat{Y}\left( ⅆ_{i} \right)= \frac{\sum_{j=1}^{n} S_{d}\left( ⅆ_{i},ⅆ_{j} \right)\times Y\left( ⅆ_{j} \right)}{\sum_{j=1}^{n} S_{d}\left( ⅆ_{i},d_{\dot{j}} \right)}$ (2)

We calculated average of the forecasts from the drug and the target to gain the ultimate estimates.

**Similarity rank-based Predictor**

Similarity Rank-based Predictor (SRP) [^19^](#_ENREF_19) calculates two tendency guides an individual drug-target pair: for the possibility that it would interact and for the possibility of no interaction respectively. The ‘Tendency-to-interact’ index for drug $ⅆ_{i}$and target $t_{j}$ is calculated as:

$TI^{+}\left( ⅆ_{i},t_{j} \right)= \sum_{p\in P^{+}\left( t_{j} \right)} \frac{S_{d}\left( ⅆ_{i},ⅆ_{p} \right)}{R_{d}\left( ⅆ_{i},ⅆ_{p} \right)}$ (3)

Wher

$$P^{+}\left( t_{j} \right) \subset\{d_{1},d_{2},\cdots,ⅆ_{n}\}$$

represent the set of drugs that tends to interact with $t_{j}$, and $R_{d}\left( ⅆ_{i},ⅆ_{p} \right)$ gives the similarity rank of the drug $ⅆ_{i}$ to $ⅆ_{p}$ among the n drugs. Another ‘Tendency-to-not-interact’ index for drug $ⅆ_{i}$and target $t_{j}$ is calculated as:

$TI^{-}\left( ⅆ_{i},t_{j} \right)= \sum_{q\in Q^{-}\left( t_{j} \right)} \frac{S_{d}\left( ⅆ_{i},ⅆ_{q} \right)}{R_{d}\left( ⅆ_{i},ⅆ_{q} \right)}$ (4)

where$Q^{-}\left( t_{j} \right)$ represents a group of drugs that tends not to intermingle with $t_{j}$. Now, the interaction possibility value is calculated as the odds relation:

$C\left( ⅆ_{i},t_{j} \right)= \frac{TI^{+}\left( ⅆ_{i},t_{j} \right)}{TI^{-}\left( ⅆ_{i},t_{j} \right)}$. (5)

The above-mentioned score is calculated by using $S_{d}$. In addition to this score, a similar corresponding score using $S_{t}$ is calculated, and then the final prediction score have been calculated by averaging both the scores.

**Bipartite local models (BLM)**

BLMs performs predictions one from the drug side and the other from the target side (i.e. BLMs performs two sets of predictions), and the scores of both the set of predictions are combined to get the ultimate estimate score for the probable communication candidate.

**SVM-based BLMs**

This revolutionary effort [^19^](#_ENREF_19)presented the idea of BLM in which a resident model is skilled to predicts, which drug would interact with which target. In the case of [^20^](#_ENREF_20), the resident models were SVM classifiers. And finally, the forecasts from both the drug and target side are averaged for the ultimate predictions.

Specifically, by assumingabipartiteDTInetwork,thealgorithm tries the prediction of edge *e_ij_*between drug *d_i_*and target *t_j_*. The following steps areperformed:

1. First ignoring *t_j_*, on the basis of known interactions of drugs with other targets (positive examples) and unknown interactions of drug *d_i_*(negative examples) a classifier is trained for *d_i_*. Interactions are labelled as +1, whereasnon-interactions are considered as -1. The skilled classifier is castoff to forecast *e_ij_*.
2. By ignoring *d_i_*, on the basis of known interactions of targets with other drugs (positive examples) and that of unknown interactions of target *t_j_*(negative examples) a classifier is trained for *d_i_*. Interactions are labelled as +1, whereasnon-interactions are considered as -1. The skilled classifier is castoff to forecast *e_ij_*.
3. Using the max (·, ·)function, estimates from both the drug and targets (i.e. from both classifiers) are combined.

Laplacian Regularized Least Squares (LapRLS) [^59^](#_ENREF_59) is an other process which is based on BLMs idea. The local models in the LapRLS generally use normalized least squares tominimizean objective function that comprises an error term along with the graph normalization term. From the drug side, the objective function is to be lessened as:

min(ǀǀY -S_d_α_d_ǀǀ_F_^2^  + β_d_T_r_(α_d_^T^S_d_L_d_S_d_α_d_)) (6)

where ǀǀ^.^ǀǀ*_F_* is the Frobenius norm, *L_d_*isthe normalized Laplacian obtained by using S_d_ and β_d_ is a constraint. Note that the hint of a given medium *A* is Tr(*A*) = Σ_i_*A_ij_*, and the expression T_r_(α_d_^T^S_d_L_d_S_d_α_d_) is a graph normalization term, which assists the model to diverse that is expected to lie underneath the information. The manifold statements (i.e. the data points are lying on a low dimensional nonlinear manifold) which are found to be usually true [^42^](#_ENREF_42) and, therefore, the enhancement of prediction performance is based on modelling the manifold. After we get α_d_^*^ which reduces the given function, the forecast matrix gained from the drug side is:

$\hat{Y}=\frac{\hat{Y}_{d}+\hat{Y}_{t}}{2}$ (7)

**Regularized least squares**

In Regularized Least squares (RLS-avg) [^22^](#_ENREF_22), the prediction is performed by the use of kernel ridge regression. Moreover, in comparison to previous approaches, Gaussian interaction profile (GIP) kernels are castoff to compute grid resemblance matrices for drugs and targets from the linking matrix Y; in order to calculate network similarity between two drugs d_i_ and d_j_ given as:

$$GIP_{d}\left( ⅆ_{i},d_{j} \right)=exp(-\gamma\|Y\left( ⅆ_{i} \right)-Y\left( ⅆ_{j} \right){\|}^{2})$$

where γ is a parameter and Y(d_i_) and Y(d_j_) are known as the interaction profiles of d_i_ and d_j_ respectively. The network similarity matrices merged with S_d_ and S_t_ as:

$K_{d}=\propto S_{d}+$ (1 – α)GIP_d_ (8) *K*_t_ $= \propto S$_d_ + (1 − α)GIP_t_ (9)

where α is a limitation such that $0\leq\alpha\leq1.$

K_d_ is a drug kernel which is designed via the linear combination of the drug network resemblance matrix GIP_d_ & the drug chemical similarity matrix S_d_, K_t_ is a target kernel which is designed via the linear combination of the target network resemblance matrix GIP_t_ and the target sequence similarity matrix S_t_. The incorporated network info (from the DTI network) was conjectured by the authors into the prediction process which leads a better performance of the prediction. After that, matrix for the prediction scores can be written as:

$\hat{Y}=\frac{1}{2}(K_{d}(K_{d}+ \sigma I)^{-1}$Y) + $\frac{1}{2}(K_{t}(K_{t}+ \sigma I)$^-1^Y^T^)^T^ (10)

Here equation (10) gives the final scores which are the average of predictions from both the target side and the drug. σ denotes the regularization parameter

In some time, a new algorithm was added in [^22^](#_ENREF_22), named RLS-kron, where the predictions from the drug and the target side are merged into one by using the Kronecker product. Given that *K = K_d_* $\otimes$*K_t_*  is a kernel over drug-target pairs, the prediction scores matrix can be given as:

$vec\left( \hat{Y}^{T} \right)=K\left( K+ \sigma I \right)$^-1^$vec\left( Y^{T} \right)$, (11)

where$vec\left( \hat{Y}^{T} \right)$ is a column vector obtained by stacking the columns of *Y^T^*. We know that the memory required by the matrix  *K* is very much, also the calculation of *K* inverse is intensive one, We used a better and more efficient [^43^](#_ENREF_43) implementation which depends upon the eigen decompositions.

**Bipartite local models**

BLMs algorithms making full use of local model have achieved a good enough performance for DTI prediction. However, the BLM has a greater issue that they don’t have the ability to train local models for drugs and targets so they don’t predict any new (drugs or targets) interactions. In order to remove this problem, Bipartite Local Models which are having Neighbor-based Interaction (BLM-NII) Profile Inferring [^23^](#_ENREF_23), based on RLS-avg, provides a pre processing technique which is represented as NII to deduce provisional interaction profiles these new targets and drugs.

Therefore, a resident model is used for each drug *d_i_*. Buj in case, if *d_i_* drug has any empty interaction profile, then a provisional linking outline will be deduced for it as given below:

$Y\left( ⅆ_{i} \right)$ *=* $\frac{\sum_{J=1}^{n} S_{d}\left( ⅆ_{i},ⅆ_{j} \right)\times Y\left( ⅆ_{j} \right)}{\sum_{J=1}^{\dot{n}} S_{d}\left( ⅆ_{i},ⅆ_{j} \right)}$ (12)

After this, the eqn (12) is normalized by using min-max normalization:

$\tilde{Y}\left( ⅆ_{i} \right)=\frac{Y\left( ⅆ_{i} \right)-\min\left( Y\left( ⅆ_{i} \right) \right)}{\max\left( Y\left( ⅆ_{i} \right) \right)-\min\left( Y\left( ⅆ_{i} \right) \right)}$ (13)

Now the drug d_i_ have an interaction profile so that the classifier training and forecast process can progress as a usual process. The NII method in a similar manner is applied to the target side wherever applicable and forecasts from the target side are also obtained. Thus, at last, the average of predictions from the drug side and target side is taken for the final score as done in the algorithms from the group of BLMs. NII pre processing procedure performance is observed better.

**Regularized least squares with weighted nearest neighbors**

The other technique which is founded on RLS-Kron [^22^](#_ENREF_22)was introduced in [^44^](#_ENREF_44)where the performance of RLS-iron was increased with a preprocessing technique WNN having the same use as that of NII. WNN can be used to deduce an interaction profile for every new drug *d_i_*,:

$Y\left( ⅆ_{i} \right)=\sum_{J=1}^{n} \omega_{j}Y\left( ⅆ_{j} \right)$, (14)

On the basis of similarity to drug d_j,_ the drugs d_i_ to d_n_ are arranged in descending order and $\omega_{j}=\eta^{j-1}$ where $\eta$ denotes the decay term and $\eta\leq$1. This procedure is applied from the target side also, and then the RLS-kron method ia used as usual process. By applying the WNN method with NII, the prediction performance boost up which shows that these preprocessing methods are doing well.

**Network Diffusion**

The methods to predict new interactions involes graph-based techniques to falls in the network diffusion category. It is named as such because this predominates in this category.

**Network-based inference**

Network-based inference (NBI) [^60^](#_ENREF_60) applies network diffusion on the DTI bipartite network corresponding to the linkage matrix *Y* to perform predictions. The working of network diffusion follows:

$\hat{Y}=WY,$ (15)

Where $W\in\mathbb{R}^{n\times n}$is the weight matrix can be defined as:

$W_{ij}=\frac{1}{\Gamma_{\left( i,j \right)}}\sum_{l=1}^{m} \frac{Y_{il}Y_{jl}}{k\left( t_{l} \right)}$ (16)

where$\Gamma$ is the diffusion rule. Whereas, k(x) denotes the degree of node i.e., x in the DTI bipartite network. While in the NBI case, the $\Gamma$ rule is given by:

$\Gamma=k\left( ⅆ_{j} \right)$.

**Heterogeneous graph inference**

This method provides an extension to NBI as presented in [^25^](#_ENREF_25). Network diffusion is based on the heterogeneous network instead of the basic bipartite network. The heterogeneous network increases the basic bipartite network by the addition of all the pairs of targets and drugs whose the edges whose weights refer to the pairwise similarities as indicated in S_d_ or S_t_, respectively. The following equation is used for network diffusion in this method.

$Y^{\left( ⅈ+1 \right)}=\alpha S_{d}Y^{\left( ⅈ \right)}S_{t}+\left( 1-\alpha\right)Y^{\left( 0 \right)}$, (17)

where$Y^{\left( ⅈ \right)}$ is the forecast value matrix at time step *i*, $Y^{\left( 0 \right)}=Y$, and *α*is an adjustable parameter. For ensuring the convergence of the formula, *S_d_*and *S_t_* are normalized as:

$S_{d}\left( ⅆ_{i},ⅆ_{j} \right)=\frac{S_{d}\left( ⅆ_{i},ⅆj \right)}{\sqrt{\sum_{k=1}^{n} S_{ⅆ}\left( d_{i},d_{k} \right)\sum_{k=1}^{n} S_{d}\left( d_{k},d_{j} \right)}}$ (18)

$S_{t}\left( t_{i},t_{j} \right)=\frac{S_{t}\left( t_{i},tj \right)}{\sqrt{\sum_{k=1}^{n} S_{t}\left( t_{i},t_{k} \right)\sum_{k=1}^{n} S_{t}\left( t_{k},t_{j} \right)}}$ (19)

**Network-based Random Walk**

Network-based Random Walk with Restart on the Heterogeneous Network (NRWRH) [^26^](#_ENREF_26) performs predictions using a heterogeneous network and having a random walk on it. NRWRH performs a random walk by using the transition matrix:

***M*** = [ $\begin{matrix} M_{DD} & M_{DT} \\ M_{TD} & M_{TT} \end{matrix}$] (20)

where$M_{DD}$and $M_{TT}$ are the changing matrices between drugs and the targets itself respectively. The $M_{TD}$ and$M_{DT}$are the transition matrices from targets to drugs and drugs to targets respectively. It may be noted that $M_{DD}$= $S_{d}$, $M_{TT}$ = $S_{t}$, $M_{DT}$ = $Y$ and $M_{TD}$ = $Y^{T}$. At step $ⅈ+1$, the predicted matrix is demonstrated as:

$A^{\left( ⅈ+1 \right)}=\left( 1-r \right)M^{T}A^{\left( ⅈ \right)}+rA^{\left( 0 \right)}$ (21)

where$A^{\left( 0 \right)}\in\mathbb{R}^{\left( n+m \right)\times\left( n+m \right)}$ is the adjacency matrix between the nodes (with the n drugs and m targets arranged in both the rows and the columns), and r denotes the restart probability. The above equation is run multiple times until the convergence is achieved, now the matrix *A* contains the scores of final predictions. And then from *A* the prediction score matrix $\hat{Y}\in\mathbb{R}^{n\times m}$ is extracted.

**Probabilistic soft logic**

With respect to previously discussed diffusion models, Probabilistic Soft Logic (PSL) [^27^](#_ENREF_27) also takes in the role a varied network alike to the one shown in Figure 2. PSL makes the use of probabilistic soft logic to carry out the forecast, i.e. it applies the use of logical connectives, such as ∧ (and), ∨ (or), and $\neg$ (not). Triads and tetrad relations (i.e. paths of length 3 and 4 respectively) between the drug and the target are searched in a heterogeneous network to determine whether the drug and target interact or not and also the forecast of possible communication is done. Triad rules are in the form of:

$SimilarTarget\left( t_{1},t_{2} \right) \wedge Interacts\left( d,t_{1} \right)$ (22)

$$\to Interacts\left( d,t_{2} \right)$$

$SimilarDrug\left( d_{1},d_{2} \right) \wedge Interacts\left( d_{1},t \right)$ (23)

$$\to Interacts\left( d_{2},t \right)$$

while tetrad rules are in the form:

$SimilarDrug\left( d_{1},d_{2} \right) \wedge SimilarTarget\left( t_{1},t_{2} \right)$ (23)

$$\wedge Interacts\left( d_{1},t_{1} \right) \to Interacts\left( d_{2},t_{2} \right)$$

The above-mentioned guidelines are applied on the DTI network wherever applicable to predict new interactions (i.e. each of the guidelines are applied to its consistent affairs existing in the network). Moreover, to evade the investigation of a big amount of traid and tetrad relations, a method known as blocking is practiced in advance where the edges corresponding to pairwise similarities between pairs of drugs and targets are removed from the network if they have weight under an approximate predefined cut off.

**Determine All Simple Paths, Find Interactions**

This method DASPfind [^28^](#_ENREF_28) predicts interaction by finding simple paths between drug and targets (i.e. having no cycles) and connect them on the varied network. To find simple tracks, each track p has its value $s_{P}$ which is calculated by multiplying the weight on the edges. At last, all the points are added up to yield the ultimate forecasts value for $\left( d,t \right)$ as per the equation:

$score= \sum_{P=1}^{z} \left( s_{P} \right)^{\alpha\times len\left( p \right)}$ (24)

where z denotes the amount of simple paths between drug d and target t, $\propto$ is adaptable decline parameter and $len\left( p \right)$ denotes the length of the path p (i.e. the contribution by the longer path to prediction score is less). It may be noted that $len\left( p \right)\leq3$. Like the PSL, the blocking procedure (i.e. the edges corresponding to pairwise similarities between pairs of drugs and targets are removed from the network if they have a weight below some user-defined cut off value) is utilized prior to forecast.

**Matrix Factorization**

Matrix factorization takes an input matrix, by using which finds two other matrices which are multiplied together and the input matrix is estimated. Considiering the present condition of DTI forecast, the linking matrix $Y\in\mathbb{R}^{nXm}$ is factorized into two matrices $A\in\mathbb{R}^{n\times k}$ and $B\in\mathbb{R}^{m\times k}$ such that $AB^{T}\approx Y$. $k$is an adjustable parameter denoting the amount of dormant characteristics in A and B, and $k \ll n,m$.

Matrix factorization recognizes the hidden features of drugs and targets in an unofficial manner, which is valuable for collaborative filtering. For example, if the hidden vectors of two drugs results to be similar, then it might be possible that these drugs share similar interactions, thus interactions between them can take place.Since we are finding absent interactions in the matrix *Y*, matrix factorization can be used as a matrix completion technique (i.e. the above-mentiocned interactions of drugs between themselves and targets themselves), which turn it out to be more significant for the DTIprediction problem.

**Kernelized Bayesian Matrix Factorization with Twin Kernels**

Kernelized Bayesian Matrix Factorization with Twin Kernels (KBMF2K) [^61^](#_ENREF_61) in our view, is the first method to use matrix factorization for the prediction of DTIs. It employes a Bayesian probabilistic design along with the concept of matrix factorization to complete the forecast. In other words, nonlinear dimensionality reduction is performed by the use of variational approximation and, hence the efficiency of computation time taken by this method is improved.The algorithmic details of this method are very broad, so a negligible impression of the algorithm is provided here [^61^](#_ENREF_61).

**Probabilistic Matrix Factorization**

Probabilistic Matrix Factorization (PMF) [^29^](#_ENREF_29) is another type of matrix factorization method that uses probabilistic formulations.

Mainly, the interactions are modeled via ‘probabilistic linear models with Gaussian noise’. Unlike KBMF2K, for prediction, it is not dependent on or uses the similarity matrices between drugs and targets, and thus it maintains relatively lower performance than the other factorization techniques mentioned here.

To understand the overall concept on the back of PMF, let us consider we have two matrices $A$ and $B$ that contains the drugs and targets latent feature vectors and that constructs the matrix $Y=AB^{T}$. The conditional possibility over detected connections in $Y$ is given by:

$p\left( Y | A,B,\sigma^{2} \right)= \prod_{i=1}^{n} \prod_{j=1}^{m} \left| f\left( \left. Y_{ij} \right|a_{i}b_{j}^{T},\sigma^{2} \right) \right|^{I_{ij}}$ (25 )

where n and m are the numbers of drugs and targets respectively, $f\left( \left. X \right|\mu,\sigma^{2} \right)$ is the Gaussian dispersed possibility density function for $x$ with mean $\mu$ and variance $\sigma^{2}$ and $I_{ij}$ is a pointer function that is equivalent to 1 if $Y_{ij}$ is recognized and otherwise 0. Considering Zero-mean, spherical Gaussian prior to the latent vectors of $A and B$, the formula of the log-likelihood of $A and B$ is resulted using Baye’s rule as:

$\ln\left( p\left( A,B | Y,\sigma^{2},\sigma_{A}^{2},\sigma_{B}^{2} \right) \right)= -\frac{1}{2\sigma^{2}}\sum_{i=1}^{n} \sum_{j=1}^{m}I_{i}j\left( Y_{ij}-a_{i}b_{j}^{T} \right)^{2}-\frac{1}{2\sigma_{A}^{2}}\sum_{i=1}^{n} a_{i}a_{i}^{T}- \frac{1}{2\sigma_{B}^{2}}\sum_{j=1}^{m} b_{j}b_{j}^{T}$ (26)

The term on the right side of the given equaion is the squared-error function which is to be lessened, though the fianal two terms, in the last, are additional Tikhonov regularization terms which are sum up for assisting in to avoid overfitting via the prevention of dormant characteristics of $A and B$ for assuming greater values. The chief purpose here is to discover the two dormant matrices $A and B$ that enhance the log-likelihood given above. At last, the ultimate forecast score matrix is gained as $\hat{Y}=AB^{T}.$

**Collaborative Matrix Factorization**

Collaborative Matrix Factorization (CMF) ^[30](#_ENREF_30)^ practices cooperative filtering for forecasting. The key purpose of matrix factorization is to discover two matrices $A and B$where $3AB^{T}\approx Y$, CMF proposes regularization terms to guarantee that$AA^{T}\approx S_{d}$and$BB^{T}\approx S_{t}$. The objective function for CMF is given by: $\underset{A,B}{\mathrm{Min}}\left\| W\otimes\left( Y-AB^{T} \right) \right\|_{F}^{2}+ \lambda_{l}\left( \left\| A \right\|_{F}^{2}+\left\| B \right\|_{F}^{2} \right)+$

$\lambda_{d}\left\| S_{d}-AA^{T} \right\|_{F}^{2}+ \lambda_{t}\left\| S_{t}-BB^{T} \right\|_{F}^{2}$ (27)

where$\left\| . \right\|_{F}$ is the Frobenius norm, $\otimes$ is the elementwise product, $\lambda_{l},\lambda_{d} and \lambda_{t}$ are parameters and $W\in\mathbb{R}^{n\times m}$is weight matrix where $W_{ij}=0$for unknown drug target pairs, so that in the estimation of $A and B$ they have no role. The first line is the weighted low-rank approximation that tries to reconstruct $Y$ by finding the latent feature matrices $A and B.$The second line is the Tikhonov regularization term that provides simpler solutions by preventing the larger values and helps in avoiding overfitting. The 3^rd^ and 4^th^ ranks are normalization terms that require latent feature vectors of similar drugs/targets to be similar and latent feature vectors of unlike drugs/targets to be dissimilar correspondingly.

MSCMF is another variant of CMF which involve using multiple similarities for both the drug and the target. Rather than the chemical structure similarity and genomic sequence similarity that istypically used for the drugs and targets respectively, ATCsimilarityisalsousedfordrugs,andGOandPPInetworksimilaritiesare used for targets. The MSCMF objective function is given as:

$$\min_{A,B}\left\| W\otimes\left( Y-AB^{T} \right) \right\|_{F}^{2}+ \lambda_{l}\left( \left\| A \right\|_{F}^{2}+\left\| B \right\|_{F}^{2} \right)+ \lambda_{d}\left\| \sum_{k=1}^{M_{d}} \omega_{d}^{k}S_{d}^{k}-AA^{T} \right\|_{F}^{2}\boldsymbol{+}$$

$\lambda_{t}\left\| \sum_{k=1}^{M_{t}} \omega_{t}^{k}S_{t}^{k}-BB^{T} \right\|_{F}^{2}+ \lambda_{\omega}\left( \left\| \omega_{d} \right\|_{F}^{2}+\left\| \omega_{T} \right\|_{F}^{2} \right)$ (28)

s.t. $\left| \omega_{d} \right|=\left| \omega_{t} \right|=1$ where $M_{d} andM_{t}$ represents the number of drugs and targets’ similarity matrices respectively, and $\lambda_{\omega}$ is a parameter. The $\omega_{d} and \omega_{T}$are the weight vectors for the linear combination of similarity matrices of drugs and targets respectively. Tikhonov regularization terms for $\omega_{d} and \omega_{T}$, while the sixth term is a restriction that ensures that weight of $\omega_{d} and \omega_{T}$ sum upto 1.

**Weighted Graph Regularized Matrix Factorization**

Weighted Graph Regularized Matrix Factorization (WGRMF) [^31^](#_ENREF_31) is similar to CMF except that it practices chart normalization terms to learn a manifold for label propagation. The objective function for WGRMF is given as:

$mⅈn_{A,B}\left\| W\otimes\left( Y-AB^{T} \right) \right\|_{F}^{2}+ \lambda_{l}\left( \left\| A \right\|_{F}^{2}+\left\| B \right\|_{F}^{2} \right)+ \lambda_{d}T_{r}\left( A^{T}\tilde{l}_{d}A \right)+ \lambda_{t}T_{r}\left( B^{T}\tilde{l}_{t}B \right)$(29)

where$T_{r}\left( . \right)$ is the trace of the matrix, and $\tilde{l}_{d} and \tilde{l}_{t}$ are the normalized graph Laplacians which are obtained from $S_{d}{and S}_{t}$ respectively. $S_{d}{and S}_{t}$are sparsified before calculating the Laplacians graph via having only a pre-selcted value of closed neighbors for individual drug and its target respectively. For more details on graphical regularization please refer to [^45^](#_ENREF_45)^,^[^46^](#_ENREF_46).

The role of the weight matrix is the same as in the CMF; we can control that unknown drug-target pair don’t contribute to interactions’ prediction by setting $W_{ij}=0$. The weight medium is vital as or else the test cases would sum no interactions (i.e. negative instances) and have unwanted effects on the predictions.

**Neighborhood regularized logistic matrix factorization**

The idea of logistic matrix factorization (LMF) [^62^](#_ENREF_62) is used by Neighborhood Regularized Logistic Matrix Factorization (NRLMF) [^32^](#_ENREF_32) for prediction. The probability of interaction between drug $ⅆ_{i}$ and target $t_{j}$ is modelled as a logistic function:

$p_{ij}=\frac{\exp\left( a_{i}b_{j}^{T} \right)}{1+\exp\left( a_{i}b_{j}^{T} \right)}$ (30)

where$a_{i}and b_{j}$ are the latent feature vectors of $ⅆ_{i} and t_{j}$ respectively. Mostly the drug-target pair interacts on higher values of $a_{i}b_{j}^{T}$ (i.e. $p_{ij}$ tends to 1). However, to avoid the overfitting of the training data, themodelbeingtrainedisregularizedby placing spherical zero-mean Gaussian priors on the latent vectors of all drugs and targets. At last, the model is more normalized via the local neighbourhoods of the drugs and targets by the use of graph normalization. The objective function to be minimized is as follows:

$$mⅈn_{A,B}\sum_{i=1}^{n} \sum_{j=1}^{m} \left( 1+ cY_{ij}- Y_{ij} \right)ln[1+\exp\left( a_{i}b_{j}^{T} \right))]- cY_{ij}a_{i}b_{j}^{T}$$

$+\lambda_{d}\left\| A \right\|_{F}^{2}+ \lambda_{t}\left\| B \right\|_{F}^{2}$ (31)

$+ \alpha T_{r}\left( A^{T}l_{d}A \right)+ \beta T_{r}\left( B^{T}l_{t}B \right)$

where*c*,$\lambda_{d}$, $\lambda_{t}$, $\alpha$and $\beta$are constraints. The first term regarding the above equation is the LMF expression, which is improved by Tikhonov and graph normalization terms in the second and third term respectively. Tikhonov regularization provides simpler solutions with smaller values and prevents the overfitting, while graph regularization terms indirectly learn the causal manifold in the information to enhance additionally precise label proliferation within the communication matrix $Y$.

**Dual-network integrated logistic matrix factorization**

Dual-network Integrated Logistic Matrix Factorization (DNILMF) [^33^](#_ENREF_33) is an extension to the NRLMF. The network-based similarity is additionally incorporated by DNILMF in such a way as it is done in RLS-avg and RLS-kron. Unlike RLS-avg and RLS-kron all the kernels (refers to similarity matrices) earlier undergo a kernel diffusion step. The local similarity matrix for a drug or a target kernel is formed by keeping the similarities to the nearest $k$ neighbour for each drug or a target while the rest of them are rejected. Then the local similarity matrix and global similarity matrix are mixed over a number of repetitions.

Let us consider we have the target order resemblance matrix $S_{t}and the$drug chemical resemblance matrix $S_{d}$.The network similarity matrix for them are$GIP_{t} and GIP_{d}$calculated from the linking matrix $Y$ as already explained in the ‘Regularized least squares.’ Then the normalization (in-between values of individual row by row’s sum) for four matrices is done and symmetrized. The local matrices $L_{t} and L_{t}.GIP$are generated as:

Let us take the target matrix $S_{t}and GIP_{t}$as an example then,

$$L_{t}\left( i,j \right)= \left\{ \begin{aligned} \frac{S_{t}\left( ⅈ,j \right)}{\sum_{k\in N_{i}} S_{t}\left( i,k \right)},j\in N_{i} \end{aligned} \right.$$

$0, otherwise$ (32)

$$L_{t.GIP}\left( i,j \right)= \left\{ \begin{aligned} \frac{{GIP}_{t}\left( ⅈ,j \right)}{\sum_{k\in N_{i}} {GIP}_{t}\left( i,k \right)},j\in N_{i} \end{aligned} \right.$$

$$0, otherwise$$

where$N_{i}$ denotes the nearest neighbours of the target $t_{j}$, and $k$ is a parameter for considering the nearest number of neighbours. Referring to the above equations, the targets which are outside the similarity list of neighbours are set to 0. The global matrices $S_{t}and GIP_{t}$ are updated on the basis of $L_{t} and L_{t}.GIP$ as:

$$S_{t}^{\left( h+1 \right)}=(L_{t})GIP_{t}^{\left( h \right)}\left( L_{t} \right)^{T}$$

${GIP}_{t}^{\left( h+1 \right)}=(L_{t.GIP})S_{t}^{\left( h \right)}\left( L_{t.GIP} \right)^{T}$ (33)

where$S_{t}^{\left( h+1 \right)} and {GIP}_{t}^{\left( h+1 \right)}$are the present matrices afterward h repetitions. Referring to the above equations, the mixing operations are taking place in parallel. After a sufficient amount of repetitions, the ultimate target resemblance matrix $K_{t}$ is gained which is the average of $S_{t}^{\left( h+1 \right)} and {GIP}_{t}^{\left( h+1 \right)}$. To find the final drug similarity matrix $K_{d}$, we follow the same method.The objective function used for predictions is:

$mⅈn_{A,B}\sum_{i_{j}} ((1+cY_{ij}- Y_{ij})ln[1+exp(\alpha AB^{T}+ \beta K_{d}AB^{T}+\gamma K_{T}AB^{T})]- cY_{ij}(\alpha AB^{T}+ \beta K_{d}AB^{T}+\gamma K_{T}AB^{T}))+ \frac{\lambda_{d}}{2}\left\| A \right\|_{F}^{2}+\frac{\lambda_{t}}{2}\left\| B \right\|_{F}^{2}$ (35)

which is based on the modified logistic function:

$p=\frac{\exp(\alpha AB^{T}+ \beta K_{d}AB^{T}+\gamma K_{T}AB^{T})}{1+exp(\alpha AB^{T}+ \beta K_{d}AB^{T}+\gamma K_{T}AB^{T})}$, (34)

where$\alpha,\beta, \gamma,\lambda_{d} and \lambda_{t}$ are parameters. As compared to the NRLMF’s logistic function from (31), the DNILMF unites information from the similarity matrices $K_{t} and K_{d}$obtained by the mixing of kernels. The final predictions matrix $Y=AB^{T}$is obtained from the matrices $A and B$ that lessen the objective function in equation (39).

Since both the methods NRLMF and DNILMF are founded on logistic matrix factorization, so their objective functions are much more similar to each other [from equations (32) and (35)]. Though the graph regularization is used by NRLMF to make use of similarity matrices $S_{d}{and S}_{t}$ for prediction, while DNILMF uses the mixing of kernels$K_{t} and K_{d}$unite them into logistic function and use them in the objective function from equation (35).

**Feature-based classification**

Such techniques require fixed-length feature vectors to represent the pairs of drug-targets explicitly. The drug-target may also be signified via the concatenated feature vector $d\oplus t=$[$d_{1},d_{2},\ldots.,d_{p},t_{1},t_{2},\ldots..,t_{q}$] which is obtained from the drug feature vector $d=$[$d_{1},d_{2},\ldots.,d_{p}]$ and the target feature vector t = [$t_{1},t_{2},\ldots..,t_{q}]$. In addition to the feature vector, there is a label for each drug-target pair which shows whether its interaction is known which means a positive class or it is noninteractive which means it is a negative class. Various supervised machine learning methods including feature vectors and labels are developed for predicting DTIS.

It may be noted that it is usually convenient to refer noninteractions as unlabeled pairs because one is not sure about these interactions whether they are true noninteractions or not. But the main drawback of the these methods is that they assume the unlabeled pairs as true noninteractive.

**Incremental and forward feature selection**

The representation of drugs is done as in [^34^](#_ENREF_34), with a number of commonly used functional groups that are presentin drugs chemical structures. However, the the targets can be represented in the form of pseudo amino acid compositions. For the betterment of the prediction performance, a pioneering feature selection procedure is furthermore introduced by the use of a better feature set in the work presented here.

For better feature selection, the process is initiated by placing features via the lowest Redundancy Maximum Relevance (mRMR) algorithm [^47^](#_ENREF_47). Then the ranked features are under the application of Incremental feature selection, i.e. the addition of ranked feature to the chosen features set in order, one by one, until the some improvement in the forecast performance on a provisional authentication set stops. At last, filtration of the set of chosen characteristics is carried out by applying forward feature selection on it. To obtain the final predictions after completing the feature selection phase, the nearest neighbour algorithm was applied.

**Random Forest and SVMs**

Random Forest and SVMs models are used to predict interactions in [^35^](#_ENREF_35). Here the assumption is for the data that consists of $n_{d}$ drugs and $n_{t}$ targets, so the total numbers of drug-target pairs are $n_{d} \times n_{t}$ in total. It becomes challenging as it utilizes the whole data sets of all drug-target pairs as training information to skillup a cataloguing model when the dimensionality of the data is high (i.e. more than one features represents the drug-target pairs).

Drug and target features were produced via the DRAGON and PROFEAT [^63^](#_ENREF_63)packages, respectively. Drug features generated by DRAGON comprise constitutional, eigenvalue-based indices and topological descriptors and 2D autocorrections above others. While in case of target features made by PROFEAT involves CTD, autocorrelation signifers and amino acid composition and so on.

**Fuzzy** $\mathbf{K}$**-nearest neighbours**

Fuzzy $K$-nearest neighbours (Fuzzy KNN) [^36^](#_ENREF_36) constructs each training instance as it belongs to two groups (i.e. positive and negative classes) with different associated standards. For every test instance, the associated value of each of the two classes is calculated by taking a weighted average of the similarities to its nearest $K$neighbours, and the group of the training instance is decided by the highest value of the two. The targets were signified by using pseudo amino acid composition, while the drugs were signified by FP2 fingerprints which were created by the Open Babel program[^11^](#_ENREF_11).

**Decision tree ensemble with oversampling**

Decision tree is an ensemble method which is ensemble with oversampling introduced for predicting the interactions [^37^](#_ENREF_37). Drug signifiers were calculated using the Rcpi pack- age [^9^](#_ENREF_9), whereas target descriptors were created via the PROFEATWebserver[^18^](#_ENREF_18). An ensemble of decision trees is trained similar to the Random Forest as used in [^35^](#_ENREF_35), and feature sub-spacing is applied (i.e. for every decision tree a subset of the feature is randomly sampled). However, in contrast to the Random forest, which performs bagging on the same tested group of negatives, for every decision tree a different group of negatives is sampled which gives the advantage to cover the negative data better and include the maximum negative data in the training procedure. Additionally, clustering is used in the interacting classes to look for the small disjuncts that are then oversampled to strengthen them. This is carried out to address a problem within-class disproportion in the known data.

**Decision tree ensemble with dimensionality reduction**

One of an additional method same as the existing one [^37^](#_ENREF_37) is EnsemDT [^38^](#_ENREF_38). Rather than oversampling small disjuncts in the decision tree, the EnsemDT uses the concept of dimensionality reduction (i.e. the dimensionality reduction is applied to the drug and target feature vectors prior to sequencing them to form the examples. There are mainly three techniques for dimensionality reduction which were examined which are named as Singular Value Decomposition (SVD), Partial Least Squares [^48^](#_ENREF_48) and Laplacian Eigenmaps [^42^](#_ENREF_42). The dimensions reduction is the most common technique used to improve the computational efficiency (i.e. the reduction of the running time), and also helpful in the improvement of prediction performance as well.

**Rotation Forest-based Predictor of Drug-Target Interactions**

RFDT [^49^](#_ENREF_49) is another ensemble learning technique for DTIs prediction. Specifically, a variant that is based upon Rotation Forest [^50^](#_ENREF_50) was used. The set of features is arbitrarily divided into $K$ roughly equal subsets for each base classifier (is a parameter). Another way, the feature matrix $X\in\mathbb{R}^{n\times p}$is divided into $K$ submatrices such as the amount of columns in an individual submedium is around$p/K$, where n represents the number of instances, and p represents the number of features. Trapping is then performed on the training set (i.e. the training set for the current base classifier is a randomly sampled set from the training examples). In the next step, (PCA) principal component analysis is performed separately on all of the submatrices, and the resulting features calculated from all the submatrices are combined that forms a diagonal block matrix, also called rotation matrix. At the end, the rotation matrix is multiplied with the feature matrix $X$. The subsequent matrix performes like a training set along with the corresponding labels to train the base classifier. The ensemble is built by recapping the technique for all the base classifiers.

Use of the rotation matrix (which is constructed by dividing a feature vector into different $K$subsets) and bagging are the methods for introducing variation in the ensemble. Increase in variation benefits in the improvement of the prediction performance [^51^](#_ENREF_51). In this study, drugs are represented by PubChem fingerprints (i.e. an indication of presence and absence of 881 common substructures by binary vectors), while autocovariance vectors generated by using the target’s genomic sequences representing targets. In particular, (PSSM) a position-specific scoring matrix was calculated for each target by using the sequence. Afterthat, this PSSM is used to obtain autocovariance vectors for targets.

**Predicting Drug Targets with Protein Sequence**

Similar to RFDT, (PDTPS) Predicting Drug Targets with Protein Sequence [^52^](#_ENREF_52) uses PSSMs to represent targets. For PSSMs, it calculates bi-gram probabilities in place of autocovariance. To reduce the actual dimensionality of the features, PCA is applied. PDTPS use Relevance Vector Machines (RVMs) to perform prediction.

RVM [^53^](#_ENREF_53) is used as a machine learning method and it is practically equivalent to the SVM. Different from SVM, RVM uses Bayesian learning so that probability formulations can be used in the predictions. Typically, the prediction model trained with the use of RVM is sparse, it maens that it is compact and interpretable, but in terma of the output produced by the RVM, it is even better than that of SVM.

**Extremely Randomized Trees**

Extremely Randomized Tree (ER-Tree) is a method for performing prediction as in [^54^](#_ENREF_54). In regular decision tree-based ensembles, there are certain rules followed by each decision tree for (i) selection of qualities effective for the use of tree-splitting and (ii) determination of cut off points within these qualities. In the ER-Tree method, randomization is generally usually included explicitly in training process by selecting the qualities and cut off points randomly. Thus, the prediction performance is improved by the reduction of variation of the tree-based model using explicit randomization. Moreover, bagging is avoided in this process whole training set is used just to keep the inclination very low.

The $K$ qualities are chosen arbitrarily for each base classifier in ER-Tree method. Each $K$ quality has an arbitrarily generated cut off point (i.e. each quality has a determined maximum and a minimum value, $a_{\max} and a_{\min}$) where the cutoff point is generated arbitrarily from the interval $\left| a_{\min}{,a}_{\max} \right|$. The formula for the evaluation of different $K$’s is given as:

$Score\left( s,N \right)= \frac{2I_{c}^{s}\left( N \right)}{H_{C}\left( N \right)+H_{s}\left( N \right)}$ (35)

where $N$ is the current tree node (i.e. before the split $s$),$N_{L}and N_{R}$are the left and right child nodes of $N$ respectively. $H_{C}\left( N \right)$is the classification entropy at $N$, $H_{s}\left( N \right)$ is the split entropy and $I_{c}^{s}\left( N \right)$ is the common information of the split outcome and classification. Particularly, $H_{C}\left( N \right), H_{s}\left( N \right) and I_{c}^{s}\left( N \right)$ are calculated as:

$$H_{C}\left( N \right)= -\sum_{i=1}^{c} p_{i}\log_{2} p_{i}$$

$H_{s}\left( N \right)= -\left( \frac{\left| N_{L} \right|}{\left| N \right|}\log_{2} \frac{\left| N_{L} \right|}{\left| N \right|}+ \frac{\left| N_{R} \right|}{\left| N \right|}\log_{2} \frac{\left| N_{R} \right|}{\left| N \right|} \right)$(36)

$$I_{c}^{s}\left( N \right)= H_{C}\left( N \right)- \frac{\left| N_{L} \right|}{\left| N \right|}H_{C}\left( N_{L} \right)- \frac{\left| N_{R} \right|}{\left| N \right|}H_{C}\left( N_{R} \right)$$

Here, *C* denotes the number of different classes (here we have considered two classes case), and $\left| N \right|$ denotes the examples ratio for the node N. The one with maximum value selected for the repetitions is given as:

$s^{*}=arg_{s_{i}}\max_{i=1\ldots K}Score(s_{i},N)$ (37)

Now, the above given step is recursively repeated for the two child nodes, $N_{L} and N_{R}$. The repetition of the step takes place until the base classifier is trained. Thus, an ensemble is formed by repeating the procedure for all base classifier. PubChem fingerprints characterize the drugs while (Pseudo-SMR) Pseudo Substitution Matrix Representationcharacterizes the targets.

**Similarity-based Inference of Drug-TARgets (SITAR)**

In comparison to the other archetypal classification methods that all are feature-based, where drug-target pairs can be represented by features of drug & target vectors. SITAR [^39^](#_ENREF_39) represents the drug-target pair in terma of a vector of positive similarity in data. Using geometric mean, the similarity of two drug-target pairs $(d,t)$ and ($d^{'},t^{'})$ can be calculated as:

$S\left( \left( d,t \right),\left( d^{'},t^{'} \right) \right)= S_{d}\left( d,d^{'} \right).{S_{t}\left( t,t^{'} \right)}^{(1-r)}$ (38)

Here, $r$ is parameter whose value is adjustible. The calculation of above written equation may result in the feature vectors having a length which is equal to the number of repetitions. After creating the feature vectors, the prediction is performed by using logistic regression.

**The chemical substructures-protein domains correlation model**

PubChem fingerprints represent drugs and targets [^55^](#_ENREF_55)^,^[^56^](#_ENREF_56). For the extraction of drug and target features Sparse Canonical Correspondence Analysis (SCCA) is applied, when occurred together gives an indication of interaction between the target and the drug. SCCA enlarges ordinary CCA by addition of $L_{1}$ norm regularization term to make sure, the attained weight vectors are less. The SCCA tries to reduce the objective function as given below:

${max}_{\alpha,\beta}\alpha^{T}D^{T}YT\beta$ s.t. $\left\| \alpha\right\|_{2}^{2}\leq1$, $\left\| \beta\right\|_{2}^{2}\leq1$, $\left\| \alpha\right\|_{1}\leq c_{1}\sqrt{u}$, $\left\| \beta\right\|_{1}\leq c_{2}\sqrt{v}$ (39)

where $D\in\mathbb{R}^{n\times u}$ and $T\in\mathbb{R}^{n\times v}$ denotes the matrices for the drug features and target features respectively, $c_{1} and c_{2}$ are parameters that can control the level of sparsity and 0$<c_{1}<1 \& 0<c_{2}<1$. SCCA produced results of the DTIs prediction are equivalent to the results by SVM. However, the main goal of SVM is to predict but SCCA not only predicts but it also acts as an interpretable classifier that can be examined and trained for learned rules which can contain a lot of constructive perceptions. SCCA as already discussed, emphasizes on the learning weight vectors, hence becomes promising to examine the weight vectors also for biological perceptions. Also, it should be noted that the nonzero elements in the learned weight vector should be resembled the important protein domains and also the chemical structures which direct DTIs.

**SVMs and minwise hashing**

PubChem fingerprints (indicates the presence and absence of 881 common substructures by binary vectors) representing drugs as in [^55^](#_ENREF_55), while domain fingerprints (that indicates the presence and also the absence of 876 protein domains [^56^](#_ENREF_56) that are obtained from Pfam database by using binary vectors that represents targets. Consider a drug vector$ɸ(C)$ as well as protein vector $ɸ(P)$, so that the tensor product of $ɸ\left( C \right) and ɸ(P)$ gives compound-protein pair fingerprint $ɸ(C,P)$ as:

$ɸ\left( C,P \right)= ɸ\left( C \right)\otimesɸ(P)$ (40)

By the application of minwise hashing [^64^](#_ENREF_64) to the compound-protein fingerprints, dimensionality reduction is attained which changes them to compressed fingerprints so that the technique accessible can be to large sets of data.

In Linear SVM, two variants of SVM are considered, hence used as a classifier. One variant with $L_{2}$ (MH-L2SVM) regularization term and the other one involving $L_{1}$(MH-L1SVM) regularization term. Both the variants used in it, produced predictions that are very similar. But somehow, weight vector learned from the MH-L1SVM is looks more fascinating as the features excavated are less than that of MH-L2SVM. It means there are fewer features to examine the insights.

In the last, by the use of the inverse of minwise hashing operation discussed in the work described above, the weight vector learned by using the compact fingerprints has been converted into the final weight vector for original fingerprints. At last, the final weight vector can be examined for its biological understanding.

**Empirical evaluation**

Here a broader empirical evaluation among various methods has been done under three different CV settings (Drug-Target interaction, Drug and Target) [^65^](#_ENREF_65).We have repeated 10-fold CV method for five time under the above written circumstances with the use of AUPR [^66^](#_ENREF_66)^,^[^67^](#_ENREF_67) (precision-recall curve area) for ealuatiing the metric. That is, the data set (particularly the interaction data) is divided into 10 folds under the procedure of each of 10-fold CV. Some of the folds are left out of the test set which are taking turns, and the prediction performance for each fold is accessed in terms of AUPR. Then, the average of all the calculated AUPRs is done to give AUPR of 10-fold CV. The process is then repeated for 5 times. Afterthat, the average of all 10-fold AUPRs is taken for the final AUPR.

**Benchmark data set**

We have presented the data sets most commomly used in the field of DTIs prediction. Particularly, ion channels (ICs), enzymes (Es), nuclear receptors (NRs) and G protein-coupled receptors (GPCRs) are used as four diverse classes of target proteins represented by four data sets. Interaction data was excavated from the KEGG [^1^](#_ENREF_1) database. In addition, a drug similarity matrix $S_{d}$ is provided by each data set in which the pairwise similarities of the drugs are calculated through SIMCOMP [90].The similarity matrix for targets $S_{t}$ is provided in which the similarities between the targets are calculated by normalized ([Smith and Waterman, 1981](#_ENREF_69)) Smith-Waterman.

**Selected methods**

In the section ‘Methods’ we have included the subsets of the methods characterizing various classes. We have preferred the Nearest Profile and the Weighted Profile over neighbour-based methods as a baseline. From the matrix factorization methods, we have used CMF and WGRMF. Again, selecting of Wang et al.’s method from ‘Heterogeneous graph inference’. Now from BLMs, the selection of RLS-WNN, Regularized Least Squares with Weighted Nearest Neighbors is made. The selection of the methods from different categories is based on their prediction performance as they are going well in performance in their respective categories stated in the publication where they are described. Therefore, these methods are used to represent their category. However, comparison of features of different methods are conducted on the basis of another benchmark data set that is already introduced in [^37^](#_ENREF_37). All the parameters for these prediction methods have been modified to give their ideal performances. To find the ideal parameters Grid search is used.

**References**

1 Kanehisa, M., Goto, S., Sato, Y., Furumichi, M. & Tanabe, M. KEGG for integration and interpretation of large-scale molecular data sets. *Nucleic acids research* **40**, D109-D114 (2011).

2 Knox, C. *et al.* DrugBank 3.0: a comprehensive resource for'omics' research on drugs: Nucleic Acids Res. *Database issue) D1035-41* (2011).

3 Gaulton, A. *et al.* ChEMBL: a large-scale bioactivity database for drug discovery. *Nucleic acids research* **40**, D1100-D1107 (2011).

4 Kuhn, M. *et al.* STITCH 4: integration of protein–chemical interactions with user data. *Nucleic acids research* **42**, D401-D407 (2013).

5 Weininger, D. SMILES, a chemical language and information system. 1. Introduction to methodology and encoding rules. *Journal of chemical information and computer sciences* **28**, 31-36 (1988).

6 Kuhn, M., Campillos, M., Letunic, I., Jensen, L. J. & Bork, P. A side effect resource to capture phenotypic effects of drugs. *Molecular systems biology* **6** (2010).

7 Skrbo, A., Begović, B. & Skrbo, S. Classification of drugs using the ATC system (Anatomic, Therapeutic, Chemical Classification) and the latest changes. *Medicinski arhiv* **58**, 138-141 (2004).

8 Lamb, J. The Connectivity Map: a new tool for biomedical research. *Nature reviews cancer* **7**, 54 (2007).

9 Cao, D.-S., Xiao, N., Xu, Q.-S. & Chen, A. F. Rcpi: R/Bioconductor package to generate various descriptors of proteins, compounds and their interactions. *Bioinformatics* **31**, 279-281 (2014).

10 Cao, D.-S. *et al.* (ACS Publications, 2013).

11 O'Boyle, N. M. *et al.* Open Babel: An open chemical toolbox. *Journal of cheminformatics* **3**, 33 (2011).

12 Jain, E. *et al.* Infrastructure for the life sciences: design and implementation of the UniProt website. *BMC bioinformatics* **10**, 136 (2009).

13 Ashburner, M. *et al.* Gene ontology: tool for the unification of biology. *Nature genetics* **25**, 25 (2000).

14 Emig, D. *et al.* Drug target prediction and repositioning using an integrated network-based approach. *PLoS One* **8**, e60618 (2013).

15 Zong, N., Kim, H., Ngo, V. & Harismendy, O. Deep mining heterogeneous networks of biomedical linked data to predict novel drug–target associations. *Bioinformatics* **33**, 2337-2344 (2017).

16 Cannataro, M., Guzzi, P. H. & Veltri, P. Protein-to-protein interactions: Technologies, databases, and algorithms. *ACM Computing Surveys (CSUR)* **43**, 1 (2010).

17 Klingström, T. & Plewczynski, D. Protein–protein interaction and pathway databases, a graphical review. *Briefings in bioinformatics* **12**, 702-713 (2010).

18 Zhang, P. *et al.* A protein network descriptor server and its use in studying protein, disease, metabolic and drug targeted networks. *Briefings in bioinformatics* **18**, 1057-1070 (2016).

19 Shi, J.-Y. & Yiu, S.-M. in *2015 IEEE International Conference on Bioinformatics and Biomedicine (BIBM).* 1636-1641 (IEEE).

20 Bleakley, K. & Yamanishi, Y. Supervised prediction of drug–target interactions using bipartite local models. *Bioinformatics* **25**, 2397-2403 (2009).

21 Xia, Z., Zhou, X., Sun, Y. & Wu, L. in *The Third International Symposium on Optimization and Systems Biology.* 123-131 (Citeseer).

22 van Laarhoven, T., Nabuurs, S. B. & Marchiori, E. Gaussian interaction profile kernels for predicting drug–target interaction. *Bioinformatics* **27**, 3036-3043 (2011).

23 Mei, J.-P., Kwoh, C.-K., Yang, P., Li, X.-L. & Zheng, J. Drug–target interaction prediction by learning from local information and neighbors. *Bioinformatics* **29**, 238-245 (2012).

24 Cheng, F., Zhou, Y., Li, W., Liu, G. & Tang, Y. Prediction of chemical-protein interactions network with weighted network-based inference method. *PloS one* **7**, e41064 (2012).

25 Wang, W., Yang, S. & Li, J. in *Biocomputing 2013* 53-64 (World Scientific, 2013).

26 Chen, X., Liu, M.-X. & Yan, G.-Y. Drug–target interaction prediction by random walk on the heterogeneous network. *Molecular BioSystems* **8**, 1970-1978 (2012).

27 Fakhraei, S., Huang, B., Raschid, L. & Getoor, L. Network-based drug-target interaction prediction with probabilistic soft logic. *IEEE/ACM Transactions on Computational Biology and Bioinformatics (TCBB)* **11**, 775-787 (2014).

28 Ba-Alawi, W., Soufan, O., Essack, M., Kalnis, P. & Bajic, V. B. DASPfind: new efficient method to predict drug–target interactions. *Journal of cheminformatics* **8**, 15 (2016).

29 Cobanoglu, M. C., Liu, C., Hu, F., Oltvai, Z. N. & Bahar, I. Predicting drug–target interactions using probabilistic matrix factorization. *Journal of chemical information and modeling* **53**, 3399-3409 (2013).

30 Zheng, X., Ding, H., Mamitsuka, H. & Zhu, S. in *Proceedings of the 19th ACM SIGKDD international conference on Knowledge discovery and data mining.* 1025-1033 (ACM).

31 Ezzat, A., Zhao, P., Wu, M., Li, X.-L. & Kwoh, C.-K. Drug-target interaction prediction with graph regularized matrix factorization. *IEEE/ACM Transactions on Computational Biology and Bioinformatics (TCBB)* **14**, 646-656 (2017).

32 Liu, Y., Wu, M., Miao, C., Zhao, P. & Li, X.-L. Neighborhood regularized logistic matrix factorization for drug-target interaction prediction. *PLoS computational biology* **12**, e1004760 (2016).

33 Hao, M., Bryant, S. H. & Wang, Y. Predicting drug-target interactions by dual-network integrated logistic matrix factorization. *Scientific reports* **7**, 40376 (2017).

34 He, Z. *et al.* Predicting drug-target interaction networks based on functional groups and biological features. *PloS one* **5**, e9603 (2010).

35 Yu, H. *et al.* A systematic prediction of multiple drug-target interactions from chemical, genomic, and pharmacological data. *PloS one* **7**, e37608 (2012).

36 Xiao, X., Min, J.-L., Wang, P. & Chou, K.-C. iGPCR-Drug: A web server for predicting interaction between GPCRs and drugs in cellular networking. *PloS one* **8**, e72234 (2013).

37 Ezzat, A., Wu, M., Li, X.-L. & Kwoh, C.-K. Drug-target interaction prediction via class imbalance-aware ensemble learning. *BMC bioinformatics* **17**, 509 (2016).

38 Ezzat, A., Wu, M., Li, X.-L. & Kwoh, C.-K. Drug-target interaction prediction using ensemble learning and dimensionality reduction. *Methods* **129**, 81-88 (2017).

39 Perlman, L., Gottlieb, A., Atias, N., Ruppin, E. & Sharan, R. Combining drug and gene similarity measures for drug-target elucidation. *Journal of computational biology* **18**, 133-145 (2011).

40 Tenenbaum, J. B., De Silva, V. & Langford, J. C. A global geometric framework for nonlinear dimensionality reduction. *science* **290**, 2319-2323 (2000).

41 Roweis, S. T. & Saul, L. K. Nonlinear dimensionality reduction by locally linear embedding. *science* **290**, 2323-2326 (2000).

42 Belkin, M. & Niyogi, P. in *Advances in neural information processing systems.* 585-591.

43 Raymond, R. & Kashima, H. in *Joint european conference on machine learning and knowledge discovery in databases.* 131-147 (Springer).

44 Van Laarhoven, T. & Marchiori, E. Predicting drug-target interactions for new drug compounds using a weighted nearest neighbor profile. *PloS one* **8**, e66952 (2013).

45 Gu, Q., Zhou, J. & Ding, C. in *Proceedings of the 2010 SIAM international conference on data mining.* 199-210 (SIAM).

46 Shang, F., Jiao, L. & Wang, F. Graph dual regularization non-negative matrix factorization for co-clustering. *Pattern Recognition* **45**, 2237-2250 (2012).

47 Peng, H., Long, F. & Ding, C. Feature selection based on mutual information: criteria of max-dependency, max-relevance, and min-redundancy. *IEEE Transactions on Pattern Analysis & Machine Intelligence*, 1226-1238 (2005).

48 De Jong, S. SIMPLS: an alternative approach to partial least squares regression. *Chemometrics and intelligent laboratory systems* **18**, 251-263 (1993).

49 Wang, L. *et al.* Rfdt: A rotation forest-based predictor for predicting drug-target interactions using drug structure and protein sequence information. *Current Protein and Peptide Science* **19**, 445-454 (2018).

50 Zhang, C.-X. & Zhang, J.-S. A variant of Rotation Forest for constructing ensemble classifiers. *Pattern Analysis and Applications* **13**, 59-77 (2010).

51 Zhou, Z.-H. *Ensemble methods: foundations and algorithms*. (Chapman and Hall/CRC, 2012).

52 Meng, F.-R., You, Z.-H., Chen, X., Zhou, Y. & An, J.-Y. Prediction of drug–target interaction networks from the integration of protein sequences and drug chemical structures. *Molecules* **22**, 1119 (2017).

53 Tipping, M. E. Sparse Bayesian learning and the relevance vector machine. *Journal of machine learning research* **1**, 211-244 (2001).

54 Huang, Y.-A., You, Z.-H. & Chen, X. A systematic prediction of drug-target interactions using molecular fingerprints and protein sequences. *Current Protein and Peptide Science* **19**, 468-478 (2018).

55 Yamanishi, Y., Pauwels, E., Saigo, H. & Stoven, V. Extracting sets of chemical substructures and protein domains governing drug-target interactions. *Journal of chemical information and modeling* **51**, 1183-1194 (2011).

56 Finn, R., Mistry, J., Tate, J., Coggill, P. & Heger, A. Pfam: the protein families database. Nuclei. Acids Re. (2014).

57 Tabei, Y. & Yamanishi, Y. Scalable prediction of compound-protein interactions using minwise hashing. *BMC systems biology* **7**, S3 (2013).

58 Yamanishi, Y., Araki, M., Gutteridge, A., Honda, W. & Kanehisa, M. Prediction of drug–target interaction networks from the integration of chemical and genomic spaces. *Bioinformatics* **24**, i232-i240 (2008).

59 Xia, Z., Wu, L.-Y., Zhou, X. & Wong, S. T. in *BMC systems biology.* S6 (BioMed Central).

60 Cheng, F. *et al.* Prediction of drug-target interactions and drug repositioning via network-based inference. *PLoS computational biology* **8**, e1002503 (2012).

61 Gönen, M. Predicting drug–target interactions from chemical and genomic kernels using Bayesian matrix factorization. *Bioinformatics* **28**, 2304-2310 (2012).

62 Johnson, C. C. Logistic matrix factorization for implicit feedback data. *Advances in Neural Information Processing Systems* **27** (2014).

63 Zhang, R., Xia, L. Q., Lu, W. W., Zhang, J. & Zhu, J. S. LncRNAs and cancer. *Oncology letters* **12**, 1233-1239 (2016).

64 Broder, A. Z., Charikar, M., Frieze, A. M. & Mitzenmacher, M. Min-wise independent permutations. *Journal of Computer and System Sciences* **60**, 630-659 (2000).

65 Pahikkala, T. *et al.* Toward more realistic drug–target interaction predictions. *Briefings in bioinformatics* **16**, 325-337 (2014).

66 Raghavan, V., Bollmann, P. & Jung, G. S. A critical investigation of recall and precision as measures of retrieval system performance. *ACM Transactions on Information Systems (TOIS)* **7**, 205-229 (1989).

67 Davis, J. & Goadrich, M. in *Proceedings of the 23rd international conference on Machine learning.* 233-240 (ACM).
